# Risk-based referral model to nephrologist specialist care in Stockholm

**DOI:** 10.1093/ndt/gfaf128

**Published:** 2025-07-09

**Authors:** Aurora Caldinelli, Anne-Laure Faucon, Arvid Sjölander, Roosa Lankinen, Antoine Creon, Edouard L Fu, Marie Evans, Juan Jesus Carrero

**Affiliations:** Department of Medical Epidemiology and Biostatistics, Karolinska Institutet, Stockholm, Sweden; Department of Medical Epidemiology and Biostatistics, Karolinska Institutet, Stockholm, Sweden; Department of Medical Epidemiology and Biostatistics, Karolinska Institutet, Stockholm, Sweden; Department of Medical Epidemiology and Biostatistics, Karolinska Institutet, Stockholm, Sweden; Department of Medical Epidemiology and Biostatistics, Karolinska Institutet, Stockholm, Sweden; Department of Medical Epidemiology and Biostatistics, Karolinska Institutet, Stockholm, Sweden; Department of Clinical Epidemiology, Leiden University Medical Center, Leiden, The Netherlands; Renal Medicine, Department of Clinical Science, Intervention and Technology, Karolinska Institutet, Karolinska Hospital, Stockholm, Sweden; Department of Medical Epidemiology and Biostatistics, Karolinska Institutet, Stockholm, Sweden; Division of Nephrology, Department of Clinical Sciences, Karolinska Institutet, Danderyd Hospital, Stockholm, Sweden

**Keywords:** kidney disease, kidney failure risk equation, nephrology referral

## Abstract

**Background:**

For most patients, clinical management of the early stages of chronic kidney disease is performed in primary care settings. The Kidney Disease: Improving Global Outcomes (KDIGO) 2024 guidelines recommend using a 5-year kidney failure risk equation (KFRE) of 3–5% to guide nephrologist referrals. Here, we aimed to assess the impact of adopting a risk-based referral model compared with traditional referral criteria.

**Methods:**

We conducted an observational retrospective study of adults with an estimated glomerular filtration rate (eGFR) <60 ml/min/1.73 m^2^ (Lund–Malmö equation) from the SCREAM project, a healthcare utilization cohort from Stockholm, Sweden. We evaluated the performance of the non–North American four-variable KFRE and recalibrated it to better fit our setting. KFRE thresholds were compared with traditional models—the clinical Swedish criteria and the classic KDIGO 2012 criteria—both of which are mainly based on age, eGFR and albuminuria thresholds. Sensitivity, specificity, positive and negative predictive values, reclassification matrices, net reclassification improvement and decision curve analyses were used to assess performance and clinical utility.

**Results:**

The study included 887 388 observations from 192 964 individuals. At inclusion, 49% were men, median age was 76 years and median eGFR was 54 ml/min/1.73 m^2^. During follow-up, 2624 (1.4%) progressed to KRT. The KFRE demonstrated good prediction performance, which further improved after recalibration. Both the non–North American– and SCREAM-recalibrated KFRE provided higher sensitivity and specificity than Swedish and classical KDIGO criteria. KFRE-based referral models yielded better net reclassification improvement, demonstrating superior performance in decision curve analyses. Higher thresholds (15% for the non–North American–recalibrated KFRE, 9% for the SCREAM-recalibrated KFRE) than the KDIGO recommended ones provided the best combined sensitivity and specificity. Compared with traditional referral models, implementation of a risk-based referral would decrease the number of unnecessary referrals by 23% and 25%, respectively.

**Conclusion:**

In a large northern European healthcare system, transitioning to a risk-based referral model would result in an important reduction in unnecessary referrals while maintaining a low rate of missed cases, optimizing resource utilization.

## Graphical Abstract

**Graphical Abstract:**
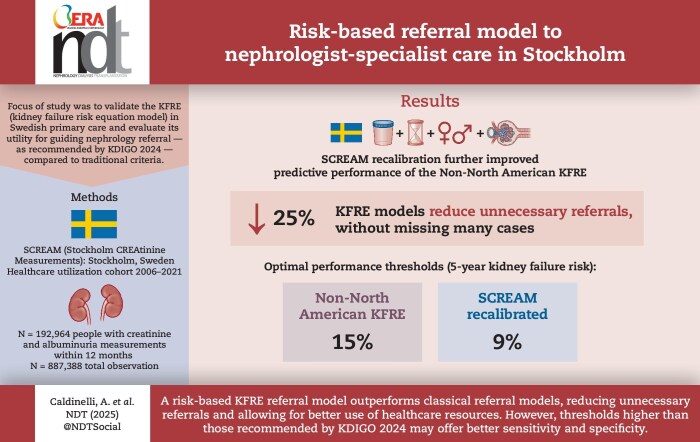

KEY LEARNING POINTS **What was known:**

- The 2024 Kidney Disease: Improving Global Outcomes (KDIGO) guidelines suggest using the kidney failure risk equation (KFRE) model for nephrology referral. Whether a risk-based model outperforms classic referral criteria is not well studied.

**This study adds:**

- KFRE-based referral model had better performance compared with classic criteria used in Sweden or those recommended by the previous 2012 KDIGO guidelines.
- The improved performance of the risk-based referral model would be achieved by reducing the number of unnecessary referrals, without missing many cases in which the referral is needed.

**Potential impact:**

- Transitioning to a risk-based referral model would result in an important reduction of unnecessary referrals while maintaining a low rate of missed cases, thus optimizing resource utilization.

## INTRODUCTION

Given the size of the population with chronic kidney disease (CKD) [1] and that, in most cases, progression to kidney failure is slow, early CKD management is often conducted in primary care [2]. Referral to a nephrology specialists, in most but not all cases, is indicated at more severe CKD stages, when loss of kidney function is very rapid or when CKD complications can no longer be adequately managed in primary care [3, 4].

Clinical guidelines provide diverse opinion-based referral criteria [5], which are usually based on specific thresholds of estimated glomerular filtration rate (eGFR) or albuminuria, and sometimes age. Minor variations in referral criteria can significantly impact

referral rates, increasing waiting times and burdening nephrology departments. Many referrals involve individuals at low risk of kidney failure progression, where specialist care may not be necessary [6].

The 2024 Kidney Disease: Improving Global Outcomes (KDIGO) guidelines recommend using a risk-based referral model with the Kidney Failure Risk Equation (KFRE), referring patients to nephrologist care when their estimated 5-year kidney failure risk is >3–5% [7]. If health systems are to transition to a risk-based referral model, it is necessary to provide a demonstration of the superiority of this model over more classic referral criteria.

This study aimed to validate and, if needed, recalibrate the four-variable KFRE equation in Swedish primary care settings, evaluate the KDIGO's suggested thresholds for the KFRE and assess the effect of implementing different risk-based thresholds for nephrology referral compared with traditional criteria.

## MATERIALS AND METHODS

### Data sources and study population

We conducted an observational retrospective study in the Stockholm CREAtinine Measurements (SCREAM) project, a healthcare utilization cohort covering all citizens in the region of Stockholm, Sweden, accessing healthcare during 2006–2021 [8]. The Swedish unique personal identification number was used to link laboratory data with regional and national administrative databases for complete information on healthcare access, diagnoses and dispensed medications.

Kidney replacement therapy (KRT) data were retrieved from the Swedish Renal Registry (SRR), a nationwide quality registry of patients with CKD referred to nephrologists in Sweden, with >97% coverage of KRT cases [9]. The regional ethical review board in Stockholm approved the study (reference 2017/793-31). The Swedish National Board of Welfare linked and de-identified the registries. Since the study uses de-identified data, informed consent was not deemed necessary.

Adults (≥18 years of age) with at least one serum or plasma creatinine and albuminuria test taken on the same date between 1 January 2006 and 31 December 2021 were included. Often in clinical practice, creatinine and albuminuria are not measured on the same day, so if the two tests were not available on the same day, a window of 12 months was considered, using the latest test date as the index date. We extracted all available pairs of creatinine and albuminuria measurements meeting this condition during the full observation period of a given patient, and thus, when available, we obtained repeated KFRE observations per patient. We excluded patients who, at cohort inclusion (first observation), had an eGFR ≥60 ml/min/1.73 m^2^, were undergoing KRT or died within 1 day. We decided to define CKD based on a single eGFR measurement because it better reflects how the KFRE is applied in routine care, where risk is calculated at each creatinine test without requiring confirmation from prior eGFR values [10]. The flow chart detailing this process is shown in Supplementary Fig. S1.

### Study exposure

The study exposures included the four-variable KFRE, current Swedish criteria for nephrologist referral [11] and the 2012 KDIGO referral criteria [12]. We intentionally disregarded referral criteria that are universally applicable regardless of KFRE, eGFR or albumin:creatinine ratio (ACR), such as rapid kidney disease progression, abnormal urine sediment, acute kidney injury, recurrent nephrolithiasis or the diagnosis of a kidney disease or genetic kidney diseases that requires specific and specialized clinical management. We also did not consider persistent CKD abnormalities such as anaemia, acidosis or bone disease [7]. Although the 2024 KDIGO guidelines [7] refer to various validated risk prediction models that could be used at the bedside, we chose to focus on the KFRE for its wide use globally and multiple external validation studies.

The four-variable KFRE, incorporating age, sex, eGFR and urine ACR, was developed in patients with CKD stages 3–5 referred to nephrology care in Ontario, Canada [13]. External validation in 31 cohorts, including the SRR, revealed variations in baseline risk, leading to a non–North American recalibration factor for improved accuracy [14]. In this study, we used this non–North American–recalibrated KFRE equation. The eGFR was calculated from serum or plasma creatinine and estimated using the revised Lund–Malmö (RLM) equation [15], as this is the validated equation automatically reported in Swedish healthcare [11] and the one with the highest precision and accuracy against measured GFR in SCREAM [16]. We considered ACR tests along with urine protein:creatinine ratio (PCR) tests and dipstick albuminuria tests that were approximated to ACR using the Sumida conversion formula incorporating comorbidities [17].

A description of the referral criteria utilized in this study is presented in Supplementary Table S1. Briefly, Swedish referral criteria [11] employ fixed thresholds of age, eGFR and albuminuria. The 2012 KDIGO criteria [12] use eGFR <30 ml/min/1.73 m^2^, significant albuminuria (ACR ≥300 mg/g) and hypertension refractory to treatment with four or more antihypertensive agents. Refractory hypertension was defined in our study as filled prescriptions for four or more antihypertensive drugs in the 6 months prior to each observation (see definitions in Supplementary Table S2). Filled prescriptions of these medications were ascertained by linkage with the national prescribed drug register [18], which has complete coverage of all dispensed prescriptions at Swedish pharmacies.

### Study covariates

A history of comorbidities and ongoing medications were defined for descriptive purposes at cohort inclusion. Comorbidities were identified using the full preceding medical history, while medications were considered ongoing if dispensed within 6 months preceding the index date. Algorithms defining study covariates through clinical diagnostic codes or pharmacy fills are detailed in Supplementary Table S2.

### Study outcome

The study outcome was KRT, defined as the date of the start of chronic dialysis or pre-emptive kidney transplantation within 5 years. During KFRE validation and recalibration we also explored a shorter horizon of 2 years. The date of KRT start was ascertained through linkage with the SRR [11].

### Statistical analysis

Descriptive statistics are represented as medians with interquartile ranges (IQRs) or numbers with percentages.

### Model discrimination, calibration and recalibration

If the KFRE is to be automatically reported in electronic healthcare records, it would be calculated every time that albuminuria or creatinine was ordered, and physicians will decide based on reported risks, not considering prior KFRE estimates or changes. To mimic this clinical practice, and to use data efficiently, we constructed one patient record for each creatinine/albuminuria measure, meeting the conditions above. Each patient record was followed from the creatinine/albuminuria measure until KRT, death or censoring, whichever came first. Censoring events were emigration from Stockholm County and the end of data collection (31 December 2021). Thus each patient contributed with multiple patient records. In the development of the original KFRE, death was considered a censoring event but not a competing event [13], which results in a systematic overestimation of the risk of KRT by assuming that people can have kidney failure after death [19]. To provide more realistic prognostic estimates, we included death as a competing risk in the validation process where feasible.

For each record, we calculated the predicted 2- and 5-year KRT risks using the four-variable KFRE. These predictions were used to evaluate the model's performance in our cohort. Model discrimination was evaluated using cumulative incidence curves, accounting for death as a competing risk, by KFRE levels [14] and using both the C-index and Brier score. We assessed calibration by plotting predicted versus observed risk to determine if predictions matched actual outcomes. The cohort was divided into 10 groups, each representing 10% of the predicted risk distribution. An additional plot was generated for the lowest 20%, as these groups are the most relevant for informing nephrology referral decisions. Observed risk within each group was calculated using a cumulative incidence function, accounting for competing risk. This allowed comparisons of KFRE model predictions with actual KRT incidence in each group.

To improve the model's performance, we recalibrated the 2-year and 5-year KFRE models using a Cox proportional hazards model fitted to our database. In the recalibration process we updated baseline hazard and regression coefficients resulting in the ‘SCREAM-recalibrated KFRE’. To retain the original KFRE structure, death was excluded as a competing risk during recalibration. The original model is referred to as the ‘non–North American KFRE’, while Swedish and KDIGO 2012 criteria are referred to together as ‘traditional referral criteria’.

### Optimal KFRE thresholds and comparison with classic nephrologist referral models

We compared the prognostic performance of the KFRE and traditional referral criteria over a 5-year horizon. Using the Youden index [20], extracted from receiver operating characteristics (ROC) curves (built including death as a competing risk), we identified optimal thresholds for both KFRE equations based on the highest sensitivity and specificity. These optimal thresholds, along with the thresholds of 3% and 5%, were compared against the traditional referral criteria.

For each referral model, we extracted pairs of sensitivity and specificity from the ROC curve. To directly compare the performance across models, we determined the sensitivity of the KFRE models at the threshold corresponding to the specificity of the traditional referral models and the specificity of the KFRE models at the thresholds corresponding to the sensitivity of the classic referral models. This allowed us to evaluate if the new criteria offered better specificity or sensitivity at equivalent levels.

### Model utility

We evaluated the clinical utility of transitioning to a risk-based KFRE model by calculating positive predictive value (PPV, true positives), false positives, negative predictive value (NPV, true negatives) and false negatives for each referral criterion. To assess whether the KFRE model improved risk prediction over traditional criteria, we computed the net reclassification improvement (NRI) [21], incorporating death as a competing risk [22]. Decision curve analysis (DCA) was used to visually compare the net benefit of referral models across various threshold probabilities [23], also considering death as a competing event.

### Sensitivity analyses

We conducted three sensitivity analyses. First, to explore if considered multiple observations per person introduced bias due to test correlation, we repeated analyses using one random observation per patient. Second, to evaluate if approximating dipstick albuminuria to ACR affected KFRE accuracy, we repeated our analyses using a cohort with ACR-only tests. The last sensitivity analysis explored whether there were differences in prognostic performance across time periods. Statistical analyses were performed using R version 4.3.1 (R Foundation for Statistical Computing, Vienna, Austria). All data have been reported in line with the TRIPOD statement (Supplementary Table S15). We used R software to develop an online calculator for the SCREAM-recalibrated model, which can be found at this link: (SCREAM Recalibrated KFRE Calculator).

## RESULTS

### Characteristics of the study population

The study included 192 964 adults with eGFR <60 ml/min/1.73 m^2^ and concomitant eGFR and ACR tests, contributing 887 388 repeated observations [median 2 per participant (IQR 1–5)]. Baseline characteristics are shown in Table 1. The median age was 76 years (IQR 69–82), 49% were men, median eGFR was 54 ml/min/1.73 m^2^ (IQR 46–57) and median albuminuria was 21 mg/g (IQR 16–54). Albuminuria was measured with ACR (including converted PCR) in 45% of cases and the remaining were dipstick tests.

**Table 1:** Descriptive characteristics at cohort inclusion (unique individuals) and of all multiple observations.

Characteristics	Patients at cohort inclusion (*n* = 192 964)	Multiple observations (*n* = 887 388)
Baseline		
Age (years), median (IQR)	76 (69–82)	77 (69–83)
Male, *n* (%)	93 788 (49)	446 980 (50)
eGFR (ml/min/1.73 m^2^), median (IQR)	54 (46–57)	47 (34–55)
Albuminuria (mg/g), median (IQR)	21.5 (15.6–53.9)	27.9 (16.9–133.1)
Type of albuminuria test, *n* (%)		
Dipstick	106 706 (55)	412 544 (46)
Urine ACR	86 258 (45)	474 844 (54)
Comorbidities, *n* (%)		
Hypertension	114 550 (59)	609 002 (69)
Diabetes	34 279 (18)	276 137 (31)
Any cardiovascular disease	61 918 (32)	360 448 (40)
Coronary artery disease	25 922 (13)	156 602 (18)
Cerebrovascular disease	16 757 (9)	97 313 (11)
Peripheral artery disease	6518 (3)	45 008 (5)
Heart failure	19 202 (10)	133 201 (15)
Drugs, *n* (%)		
Any antihypertensive agents	147 831 (77)	753 170 (85)
>3 antihypertensive agents	25 827 (13)	170 224 (19)

### Model discrimination, calibration and recalibration

**Figure 1:**
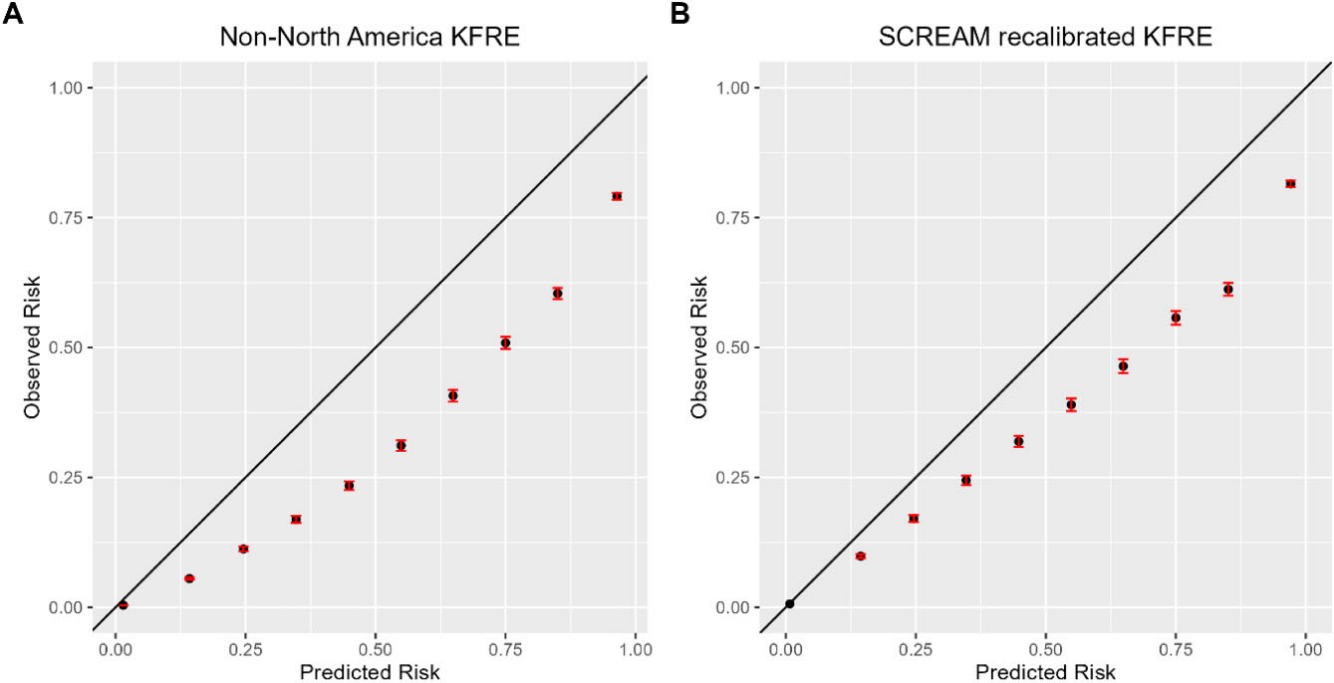
Calibration plot of expected versus observed 5-year KRT risk of the **(A)** non–North America KFRE and the **(B)** SCREAM-recalibrated KFRE. Groups are split into 10% of predicted risk. The black dots represent the predicted and observed risk for each group. The red vertical lines represent the 95% confidence intervals. The black line indicates perfect calibration.

Among the cohort, 2624 (1.4%) progressed to KRT and 76 609 (40%) died (Supplementary Table S3). The 2-year and 5-year non-North American KFRE demonstrated good discrimination, as shown by the C-index and Brier score (Supplementary Table S4) and also by Supplementary Fig. S2. Calibration plots for the 5-year KRT risk predictions are shown in Fig. 1A and the equivalent plots for the 2-year KRT risk are in Supplementary Fig. S3A. The 2-year risk model generally showed good calibration but slightly underestimated risk in lower-risk groups and overestimated it in higher-risk groups. The 5-year risk model underestimated risk across all groups.

We recalibrated the abovementioned models to better fit the Swedish setting; the derived coefficients of the resulting ‘SCREAM-recalibrated KFRE’ are provided in Appendix 1. SCREAM-recalibrated KFRE models improved calibration, but the 5-year model continued to underestimate risks (Fig. 1B and Supplementary Fig. S3B).

### Optimal KFRE thresholds and comparison with classic nephrologist referral models

Optimal 5-year KRT risk thresholds were identified using the ROC curve (Supplementary Fig. S6). A threshold of 15% for the non-North American KFRE and 9% for the SCREAM-recalibrated KFRE provided the highest sum of sensitivity and specificity, per the Youden index.

The prediction performance of these thresholds, along with the KDIGO's recommended 3% and 5%, were compared with Swedish and classical KDIGO referral models (Table 2). All referral models and all KFRE thresholds showed excellent sensitivity (ranging from 0.91 to 0.98). However, specificity varied: the classic KDIGO had the lowest specificity (0.63), while the highest specificity was observed at the optimal threshold for the non-North American KFRE (0.88 at threshold 15%) and for the SCREAM-recalibrated KFRE (0.89 at threshold 9%).

**Table 2:** Sensitivity and specificity of nephrologist referral models for prediction of 5-year KRT risk.

KFRE referral model					Traditional criteria			
	Non–North American KFRE		SCREAM-recalibrated KFRE		Swedish referral model		Classic KDIGO referral model	
Threshold	Sensitivity	Specificity	Sensitivity	Specificity	Sensitivity	Specificity	Sensitivity	Specificity
3%	0.98	0.72	0.95	0.82				
5%	0.97	0.78	0.93	0.86	0.98	0.65	0.98	0.63
9%^*^	–	–	0.91	0.89				
15%^*^	0.91	0.88	–	–				

Optimal threshold according to the Youden index.

Direct comparisons in Supplementary Table S5A show both KFRE models outperform traditional criteria, offering higher sensitivity at equivalent specificity and vice versa. For example, to achieve the same sensitivity as the Swedish referral model (0.98), the threshold of the SCREAM-recalibrated KFRE would need to decrease to 0.6%. Still, at this threshold, specificity is higher (0.70) than that achieved by the Swedish referral model (0.65, shown in Table 2). Supplementary Table S5B directly compares the two KFRE models. At the sensitivity and specificity levels of the 3% and 5% thresholds of the non-North America KFRE, the SCREAM-recalibrated KFRE shows slightly worse performance, with marginally lower specificity at fixed sensitivity and vice versa.

The classification performance of the various referral models is shown in Table 3. Classic referral models (Swedish and old KDIGO) classify more observations as meeting nephrologist referral criteria, resulting in lower PPVs and higher false positive rates. Conversely, the KFRE models classify fewer observations as eligible for referral, yielding higher PPVs, particularly at the optimal thresholds identified by ROC curve analysis. All models achieved nearly perfect NPVs (≈100%) and negligible rates of false negatives (<1%).

**Table 3:** Classification performance of different nephrologist referral models for predicting the 5-year risk of KRT.

	Observations eligible for referral			Observations non-eligible for referral		
Referral model	Observations, *n* (%)	True positives (PPV), *n* (%)	False positives, *n* (%)	Observations, *n* (%)	True negatives (NPV), *n* (%)	False negatives, *n* (%)
Swedish referral model	328 039 (37)	36 939 (11)	291 100 (89)	559 349 (63)	558 647 (99.9)	702 (0.1)
Classic KDIGO referral model	347 112 (39)	36 835 (11)	310 277 (89)	540 276 (61)	539 470 (99.9)	806 (0.1)
**KFRE referral model**						
Non-North American KFRE						
KFRE 3%	270 136 (30)	36 950 (14)	233 186 (86)	617 252 (70)	616 561 (99.9)	691 (0.1)
KFRE 5%	219 313 (25)	36 565 (17)	182 748 (83)	668 075 (75)	666 999 (99.9)	1076 (0.2)
KFRE 15%^^*^^	130 654 (15)	34 640 (27)	96 014 (73)	756 734 (85)	753 733 (99.9)	3001 (0.4)
SCREAM-recalibrated KFRE						
KFRE 3%	182 978 (21)	35 970 (20)	147 008 (80)	704 410 (79)	702 739 (99.9)	1671 (0.2)
KFRE 5%	153 683 (17)	35 391 (23)	118 292 (77)	733 705 (83)	731 455 (99.9)	2250 (0.3)
KFRE 9%^^*^^	122 395 (14)	34 348 (28)	88 047 (72)	764 993 (86)	761 700 (99.9)	3293 (0.4)

Optimal threshold according to the Youden index.

Table 4 presents the reclassification matrices comparing the Swedish and KDIGO referral models with KFRE at various thresholds. At all thresholds, both KFRE models consistently reclassify as non-eligible for referral many observations incorrectly classified by the Swedish and classic KDIGO criteria.

**Table 4:** Reclassification performance of the KFRE referral model **(A)** non–North American KFRE and **(B)** SCREAM-recalibrated KFRE versus the Swedish and classic KDIGO referral models in the prediction of 5-year risk of KRT.

	**KFRE 3%**		**KFRE 5%**		KFRE 15%^*^		
**Model**	**No referral**	**Referral**	**No referral**	**Referral**	**No referral**	**Referral**	**Total**
**Panel A. Non–North American KFRE referral model**							
Swedish referral model							
No referral	506 741 (57)	52 608 (6)	526 584 (59)	32 765 (4)	554 538 (62)	4811 (0.5)	559 349 (63)
Referral	110 511 (13)	217 528 (24)	141 491 (16)	186 548 (21)	202 196 (23)	125 843 (14)	328 039 (37)
Total	617 252 (70)	270 136 (30)	668 075 (75)	219 313 (25)	756 734 (85)	130 654 (15)	
Classic KDIGO referral model							
No referral	502 525 (57)	37 751 (4)	524 992 (59)	15 284 (2)	539 543 (61)	733 (0.001)	540 276 (61)
Referral	114 727 (13)	232 385 (26)	143 083 (16)	204 029 (23)	217 191 (24)	129 921 (15)	347 112 (39)
Total	617 252 (70)	270 136 (30)	668 075 (75)	219 313 (25)	756 734 (85)	130 654 (15)	
	**KFRE 3%**		**KFRE 5%**		KFRE 9%^*^		
**Model**	**No referral**	**Referral**	**No referral**	**Referral**	**No referral**	**Referral**	**Total**
**Panel B. SCREAM recalibrated referral model**							
Swedish referral model							
No referral	535 032 (60)	24 317 (3)	545 097 (61)	14 252 (2)	554 352 (62)	4997 (0.6)	559 349 (63)
Referral	169 378 (19)	158 661 (18)	188 608 (22)	139 431 (15)	210 641 (24)	117 398 (13)	328 039 (37)
Total	704 410 (79)	182 978 (21)	733 705 (83)	153 683 (17)	764 993 (86)	122 395 (14)	
Classic KDIGO referral model							
No referral	534 599 (60)	5677 (1)	538 168 (61)	2108 (0.002)	539 657 (61)	619 (0.001)	540 276 (61)
Referral	169 811 (19)	177 301 (20)	195 537 (22)	151 575 (17)	225 336 (25)	121 776 (14)	347 112 (39)
Total	704 410 (79)	182 978 (21)	733 705 (83)	153 683 (17)	764 993 (86)	122 395 (14)	

All values presented as *n* (%).

*Optimal threshold according to the Youden index.

### Model utility

**Figure 2:**
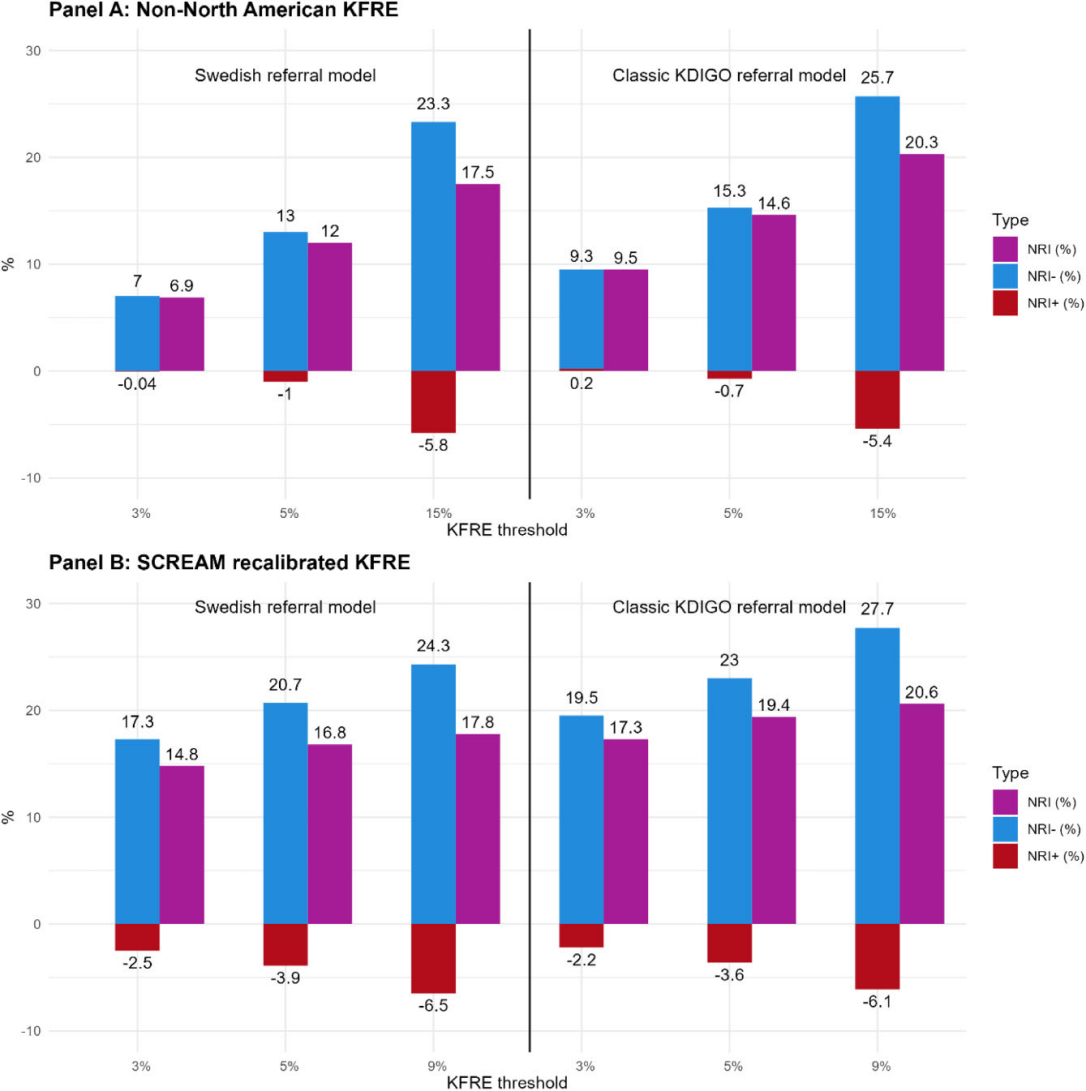
Net Reclassification Improvement (NRI) for selected thresholds of KFRE compared with the Swedish and classic KDIGO nephrologist referral models in the prediction of 5-year risk of KRT. **(A)** NRI with the non–North American KFRE and **(B)** NRI with the SCREAM-recalibrated KFRE. NRI+: NRI for events; NRI−: NRI for non-events.

The NRI are presented in Fig. 2 and Supplementary Table S6. Both KFRE models improved classification of non-events (NRI−) compared with classic referral models, meaning they are better at identifying patients who do not need referral. However, their performance in classifying events (NRI+) is slightly less accurate, indicating a minor reduction in identifying the absolute numbers of patients who need referrals. Despite this, the overall NRI supports KFRE models. For example, transitioning to a risk-based KFRE referral model using the highest threshold (15% for non-North American KFRE and 9% for SCREAM-recalibrated KFRE) would correctly reclassify 17% of observations of the Swedish referral model. This would be mainly achieved by avoiding many ‘unnecessary’ referrals (23–24%), as those patients did not progress to kidney failure within 5 years.

**Figure 3:**
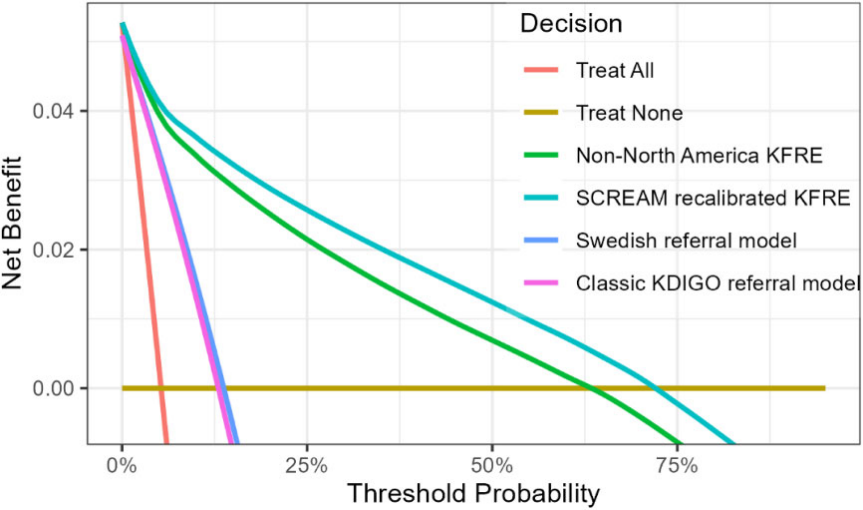
Decision curve analyses comparing differences in net benefit across different nephrologist referral models for predicting the 5-year risk of KRT. Threshold probabilities refer to the point at which a clinician would opt for treatment, so lower thresholds represent the clinical setting where the clinician is more concerned about missing true positives and therefore is willing to act even if the probability of the outcome is low. High-threshold probabilities mean that the clinician is more concerned about avoiding false positives and so the clinician will only act when there is a high probability of the outcome.

The DCA plot illustrates that KFRE models provide greater net benefit compared with traditional referral criteria across all the threshold probabilities (Fig. 3).

### Sensitivity analyses

By selecting a random observation per individual (*n* = 192 694 individuals), we observe similar results to our main analysis, with KFRE referral models outperforming traditional ones (Supplementary Table S8, Supplementary Fig. S7). Selecting only observations with ACR measurements (*n* = 474 844 observations) provided similar findings to our main analysis (Supplementary Table S11, Supplementary Fig. S8). The analysis showed consistent results across different time periods (Supplementary Table S14).

## DISCUSSION

By exploring kidney failure risk in this large cohort of CKD patients managed in primary healthcare, we provide support for the recommendation of the 2024 KDIGO guidelines to transition to a risk-based referral model [7]. Using KFRE to guide referrals would significantly impact referral patterns and healthcare resource utilization. A risk-based KFRE referral model outperforms current criteria, primarily by reducing unnecessary referrals. However, we also found that using higher KFRE thresholds than those proposed by the KDIGO would further improve the models’ performance.

In Manitoba, Canada, where the KFRE was initially developed, a KFRE risk of >3% over 5 years has been a component of the nephrology referral process in recent years. Compared with the period before the introduction of the KFRE, a study observed shorter waiting periods and thereby improved access to care for patients at the highest risk of CKD progression [24]. In the UK, two studies in primary care found KFRE thresholds >5% [25] or >3% [26] superior to the UK National Institute for Health and Care Excellence (NICE) criteria [27]. As a result, NICE changed their recommendations to encourage implementing a risk-based referral model in the UK [28]. Similar prognostic superiority of a KFRE >3% compared with Australian referral criteria was observed in a small study of 1511 patients under nephrology care [29].

Our study expands on preceding evidence with novel observations and methodological improvements. We evaluated the prognostic performance across the entire KFRE risk spectrum. A key finding is the reporting of optimal KFRE thresholds, which were markedly higher than those suggested by guidelines. The rationale for most studies and guidelines referring at the 3% and 5% thresholds is unclear, but it seems to derive from physician surveys [14] and the original KFRE study [13], conducted in a relatively small cohort of people already referred to nephrologist care with CKD stages 3b–5. It does not fully represent the population managed in primary care, in whom CKD stage 3a is more prevalent and less likely to progress to KRT within 5 years. Our findings thus show that higher KFRE thresholds naturally improve performance. Our study also benefits from utilizing repeated observations within a unified healthcare system, reducing the impact of fragmented care or unequal care access. Finally, we considered the competing risk of death during the validation process and considered all repeated measurements per individual to better approximate a real-world scenario.

Since this study was conducted in Sweden, we compared KFRE performance against current Swedish referral criteria. To generalize to a more general setting in other countries we also compared the KFRE with common referral criteria based on eGFR, albuminuria and refractory hypertension, widely used in national guidelines [5]. Regardless of the model compared, the KFRE offered improved prognostication.

We demonstrate that transitioning to a risk-based referral model would reduce the number of referrals by eliminating false positives, with the reductions in effort, time and costs that this conveys. Translating to numbers, using the non–North American optimal KFRE threshold of 15% instead of the Swedish referral criteria would decrease the proportion of referrals in the Stockholm region from 37% to 15%. Such a reduction in consultation volume is expected to decrease waiting times for high-risk patients, thereby allowing for better use of healthcare resources [24]. However, this would not be desirable if many patients progressing to kidney failure were missed. In our study, we show that using a higher KFRE threshold also increased the number of true positives (from 11% using the Swedish criteria to 27% with the optimal KFRE) with a minimal impact on false negatives, which were only 0.3% higher with the optimal KFRE referral model.

We recognize that our prognostic prediction cannot prove the real effect of its implementation. The underlying assumption for referral to nephrologist care is that patients who present late to specialty care have worse outcomes compared with patients who have a timely referral. This being said, the well-intended belief of a benefit of early versus late referral has not been proven in the form of a clinical trial, and most observational studies on this topic have focused on patients at a very high risk of end-stage kidney disease (ESKD), where the late presenters have been known to the nephrology department for <3 months before starting KRT [30]. Qualitative research [31] suggests that patients with advanced CKD desire to have prognostic information and are interested in knowing their risk of developing ESKD, and patients believe [32] that use of the KFRE in clinical decision-making would be beneficial for them.

We believe that our results can assist European policymakers in general, and Swedish ones in particular, in their decision to adopt the suggestions of the KDIGO guidelines and transition to a risk-based referral model [7]. For Sweden, we propose a recalibrated KFRE equation that could be integrated in the automatic reporting of eGFR currently available in most electronic health data systems. We also suggest adopting a KFRE referral threshold of 9%, demonstrating the best prognostic value in the Swedish setting. For other non–North American settings, we propose an optimal KFRE threshold of 15%. However, we encourage individual countries and health systems to investigate the best-fitting equations and thresholds tailored to their background risk.

Our study has limitations. Since our proposed thresholds were derived and tested on the same dataset, the results may not generalize to other regions or periods, though supporting literature strengthens their potential applicability [25, 26, 29]. Implicit in the calculation of the KFRE, we could not evaluate the utility of this model in patients with an eGFR ≥60 ml/min/1.73 m^2^. However, the 5-year risk of KRT in such patients is likely low, except perhaps for young patients with nephrotic-range proteinuria, which is per se an indication for referral to nephrology care. Moreover, numerous studies report low rates of ACR testing in people at risk of CKD, where guidelines emphasize annual screening. This continues to represent a barrier towards identification of patients in need of timely referral [33]. It is also important to note that although risk-based referral models may have benefits, they do not replace educational programs directed to primary healthcare, since there are circumstances when nephrology referral should be based on grounds other than risk.

In conclusion, transitioning from traditional criteria to a risk-based model for referrals to nephrologist care would substantially reduce the number of referrals, while improving the identification of patients at highest risk of KRT. Our findings thus support the recommendations of the 2024 KDIGO guidelines and have significant implications for patients, clinicians, policymakers and resource allocators.

## Supplementary Material

gfaf128_Supplemental_File

## Data Availability

The data underlying this article cannot be shared publicly due to the privacy of individuals who participated in the study. The data may be shared upon reasonable request for academic research collaborations that fulfil the General Data Protection Regulation as well as national and institutional ethics regulations and standards by contacting Juan-Jesus Carrero (juan.jesus.carrero@ki.se).
