# Supplementary material for: Risk-based referral model to nephrologist specialist care in Stockholm
Source: Nephrol Dial Transplant. 2025 Jul 9;41(1):102–11. doi: 10.1093/ndt/gfaf128 (PMC12722175; doi:10.1093/ndt/gfaf128)
Supplement: gfaf128_Supplemental_File [file gfaf128_supplemental_file.docx]

**SUPPLEMENTARY MATERIAL**

**Clinical utility of a risk-based referral model to nephrologist-specialist care; the Stockholm CREAtinine Measurements (SCREAM) project**

Aurora Caldinelli, Anne-Laure Faucon, Arvid Sjölander, Roosa Lankinen, Antoine Creon, Edouard L Fu, Marie Evans, Juan Jesus Carrero

**TABLE OF CONTENTS:**

[**Table S1:** Description of current referral criteria in Sweden and recommendations from the 2012 and 2021 KDIGO guidelines 3](#_Toc198825632)

[**Table S2:** Algorithms to define comorbid conditions and ongoing medications 4](#_Toc198825633)

[**Table S3:** Number of death and KRT per year of follow-up 5](#_Toc198825634)

[**Table S4:** Brier score and C-index for the Non-North America KFRE and SCREAM Recalibrated KFRE at 2 and 5 years. 6](#_Toc198825635)

[**Table S5:** Directly comparison between sensitivity and specificity of nephrologist-referral models for prediction of 5-year KRT risk 7](#_Toc198825636)

[**Table S6:** Net Reclassification Improvement (NRI) for selected thresholds of KFRE compared with the Swedish and Classic KDIGO nephrologist referral models in the prediction of 5-year risk of KRT. 8](#_Toc198825637)

[**Table S7:** Sensitivity and Specificity for Non-North America KFRE and the SCREAM recalibrated KFRE across different thresholds. 9](#_Toc198825638)

[**Table S8:** Sensitivity analysis considering one random observation per patient. Sensitivity and specificity of nephrologist-referral models for prediction of 5-year KRT risk. 10](#_Toc198825639)

[**Table S9:** Sensitivity analysis considering only one random observation per patients. Classification performance of different nephrologist-referral models for predicting the 5-year risk of KRT. 11](#_Toc198825640)

[**Table S10:** Sensitivity analysis considering only one random observation per patients. Reclassification performance of the KFRE referral model 12](#_Toc198825641)

[**Table S11:** Sensitivity analysis considering only uACR observations. Sensitivity and specificity of nephrologist-referral models for prediction of 5-year KRT risk. 13](#_Toc198825642)

[**Table S12:** Sensitivity analysis considering only uACR observations. Classification performance of different nephrologist-referral models for predicting the 5-year risk of KRT. 14](#_Toc198825643)

[**Table S13:** Sensitivity analysis considering only uACR observations. Reclassification performance of the KFRE referral model 15](#_Toc198825644)

[**Table S14:** Sensitivity analysis considering three different time periods. Sensitivity and specificity of nephrologist-referral models for prediction of 5-year KRT risk. 16](#_Toc198825645)

[**Table S15:** TRIPO checklist 17](#_Toc198825646)

[**Figure S1:** Study flowchart. 18](#_Toc198825647)

[**Figure S2:** Cumulative incidence curves of KRT events across KFRE risk groups. 19](#_Toc198825648)

[**Figure S3:** Calibration plot of expected versus observed 2-year KRT risk, considering the non-North America calibrated KFRE (panel A) and the SCREAM recalibrated KFRE (panel B). 20](#_Toc198825649)

[**Figure S4:** Calibration plot of expected versus observed 5-year KRT risk for risk<20%, considering the non-North America calibrated KFRE (panel A) and the SCREAM recalibrated KFRE (panel B). 21](#_Toc198825650)

[**Figure S5:** Calibration plot of expected versus observed 2-year KRT risk for risk<20%, considering the non-North America calibrated KFRE (panel A) and the SCREAM recalibrated KFRE (panel B). 22](#_Toc198825651)

[**Figure S6**: Time-varying Receiver Operator Characteristic (ROC) curves for the non-North America calibrated KFRE and the SCREAM recalibrated KFRE in the prediction of 5-year KRT risk. 23](#_Toc198825652)

[**Figure S7:** Sensitivity analysis considering one random observation per patient. 24](#_Toc198825653)

[**Figure S8:** Sensitivity analysis considering only uACR observations. 25](#_Toc198825654)

[**Appendix 1:** Non-north American model and SCREAM recalibrated model 26](#_Toc198825655)

### **Table S1:** Description of current referral criteria in Sweden and recommendations from the 2012 and 2021 KDIGO guidelines used in this study

| **Criteria** | **Age** | **eGFR Threshold** | **Albuminuria (ACR)** | **Other Factors** | **Referral Trigger** |
| --- | --- | --- | --- | --- | --- |
| Swedish criteria* | <75 | <45 mL/min/1.73 m² | <30 mg/mmol | - | Fixed thresholds |
|  | <75 | <60 mL/min/1.73 m² | ≥30 mg/mmol | - | Fixed thresholds |
|  | ≥75 | <15 mL/min/1.73 m² | <70 mg/mmol | - | Fixed thresholds |
|  | ≥75 | <45 mL/min/1.73 m² | ≥70 mg/mmol | - | Fixed thresholds |
| KDIGO 2012** | Any | <30 mL/min/1.73 m² | ACR ≥300 mg/g | Refractory hypertension (≥4 antihypertensives) | Clinical risk factors |
| KDIGO 2024*** | Any | Included in KFRE | Included in KFRE | Uses risk prediction via KFRE | 5-year kidney failure risk >3–5% |

* Svenskt njurregister: <https://www.medscinet.net/snr/>.
** KDIGO 2012 clinical practice guideline for the evaluation and management of chronic kidney disease. Kidney international. 2013;3:1.
*** Kidney Disease: Improving Global Outcomes CKDWG. KDIGO 2024 Clinical Practice Guideline for the Evaluation and Management of Chronic Kidney Disease. Kidney Int. 2024;105(4S):S117-S314.

*Other reasons for nephrologist referral mentioned by 2024 KDIGO guidelines and not modelled in this study are for examples: rapid kidney disease progression, abnormal urine sediment, acute kidney injury, recurrent nephrolithiasis, hereditary kidney disease or genetic kidney diseases.*

### **Table S2:** Algorithms to define comorbid conditions and ongoing medications

| **Comorbid condition** | **International Classification of Diseases (ICD) 10^th^ version codes** |
| --- | --- |
| Hypertension | I10-I15 |
| Diabetes | E10, E11 |
| Heart failure | I50, I110, I130, I132 |
| Any cardiovascular disease | I20-25, I110, I130, I132, I50, J81, I480, I481, I482, I489, G45, G46, I60-I69, I70-I73 |
| Coronary artery disease | I20-I25 |
| Cerebrovascular disease | G45, G46, I60, I61, I62, I63, I64, I65, I66, I67, I69 |
| Peripheral artery disease | I70, I72, I73 |
| **Medication** | **Anatomic Therapeutic Classification (ATC) codes** |
| ACEi | C09A, C09B |
| ARB | C09C, C09D |
| Thiazide and thiazide-like diuretics | C03A, C03B, C09BA, C09DA, C03EA |
| Loop diuretics | C03C, C03EB |
| MRA | C03DA |
| Other K-sparing diuretics (non-MRA) | C03DB, C03E |
| CCB | C08, C09BB, C09DB, C07F |
| Beta blockers | C07 |
| Alpha-blockers | C02C |
| Other antihypertensive drugs | C02A, C02DC, C09DX04 |

Abbreviations: ACEi, angiotensin-converting-enzyme inhibitor; ARB, angiotensin receptor blockers; CBB, calcium channel blockers; MRA, mineralocorticoid receptor antagonist.

### **Table S3:** Number of death and KRT per year of follow-up

| Year of follow-up | Number of deaths | Number of KRT |
| --- | --- | --- |
| 1 | 21625 | 1101 |
| 2 | 12210 | 508 |
| 3 | 9788 | 306 |
| 4 | 7770 | 203 |
| 5 | 6246 | 159 |
| 6 | 5059 | 104 |
| 7 | 3616 | 77 |
| 8 | 2923 | 45 |
| 9 | 2197 | 41 |
| 10 | 1632 | 36 |
| 11 | 1325 | 18 |
| 12 | 931 | 13 |
| 13 | 682 | 11 |
| 14 | 375 | 1 |
| 15 | 178 | 0 |
| 16 | 52 | 1 |

### **Table S4:** Brier score and C-index for the Non-North America KFRE and SCREAM Recalibrated KFRE at 2 and 5 years.

|  | ***2 years*** | | ***5 years*** | |
| --- | --- | --- | --- | --- |
|  | **Non-North America KFRE** | **SCREAM recalibrated KFRE** | **Non-North America KFRE** | **SCREAM recalibrated KFRE** |
| Brier score | 0.0154 (0.0152 – 0.0156) | 0.0145 (0.0143 – 0.0147 | 0.0349 (0.0345 – 0.0353) | 0.0306 (0.0302 – 0.0310) |
| C-index | 0.975 (0.974 – 0.976) | 0.976 (0.975 – 0.977) | 0.959 (0.958 – 0.960) | 0.960 (0.959 – 0.961) |

### **Table S5:** Directly comparison between sensitivity and specificity of nephrologist-referral models for prediction of 5-year KRT risk

***Panel A:*** *Direct comparison of performance between Swedish referral criteria/classical KDIGO referral criteria and of Non-North America KFRE/SCREAM Recalibrated KFRE*

|  | **KFRE referral model** | | | | | |
| --- | --- | --- | --- | --- | --- | --- |
|  | **Non-North American KFRE** | | | **SCREAM recalibrated KFRE** | | |
|  | Corresponding threshold | Sensitivity | Specificity | Corresponding threshold | Sensitivity | Specificity |
| **Compared to Swedish referral model given:** | | | | | | |
| Same sensitivity | 2.9% | 0.98 | 0.72 | 0.6% | 0.98 | 0.70 |
| Same specificity | 1.8% | 0.99 | 0.65 | 0.4% | 0.98 | 0.65 |
| **Compared to classic KDIGO referral model given:** | | | | | | |
| Same sensitivity | 2.9% | 0.98 | 0.72 | 0.6% | 0.98 | 0.70 |
| Same specificity | 1.6% | 0.99 | 0.63 | 0.3% | 0.99 | 0.63 |

**Panel B:** Direct comparison of performance between Non-North American KFRE referral criteria and SCREAM Recalibrated KFRE

|  | **SCREAM recalibrated KFRE** | | |
| --- | --- | --- | --- |
|  | Corresponding threshold | Sensitivity | Specificity |
| **Compared to Non-North American KFRE (3%) given:** | | | |
| Same sensitivity | 0.6% | 0.98 | 0.70 |
| Same specificity | 1.3% | 0.98 | 0.72 |
| **Compared to Non-North American KFRE (3%) given:** | | | |
| Same sensitivity | 0.8% | 0.97 | 0.77 |
| Same specificity | 1.6% | 0.96 | 0.78 |

Abbreviations: KDIGO, Kidney Disease Improving Global Outcome; KFRE, Kidney Failure Risk Equation; SCREAM, Stockholm CREAtinine Measurements.

### **Table S6:** Net Reclassification Improvement (NRI) for selected thresholds of KFRE compared with the Swedish and Classic KDIGO nephrologist referral models in the prediction of 5-year risk of KRT.

| **Non-North America KFRE** | | | |
| --- | --- | --- | --- |
| **Swedish referral model** | | | |
|  | **NRI** | **NRI+** | **NRI-** |
| **KFRE 3%** | 6.9% | -0.04% | 7.0% |
| **KFRE 5%** | 12.0% | -1.0% | 13.0% |
| **KFRE 15%*** | 17.5% | -5.8% | 23.3% |
| **Classical KDIGO referral model** | | | |
|  | **NRI** | **NRI+** | **NRI-** |
| **KFRE 3%** | 9.5% | 0.2% | 9.3% |
| **KFRE 5%** | 14.6% | -0.7% | 15.3% |
| **KFRE 15%*** | 20.3% | -5.4% | 25.7% |
| **SCREAM recalibrated KFRE** | | | |
| **Swedish referral model** | | | |
|  | **NRI** | **NRI+** | **NRI-** |
| **KFRE 3%** | 14.8% | -2.5% | 17.3% |
| **KFRE 5%** | 16.8% | -3.9% | 20.7% |
| **KFRE 9%*** | 17.8% | -6.5% | 24.3% |
| **Classical KDIGO referral model** | | | |
|  | **NRI** | **NRI+** | **NRI-** |
| **KFRE 3%** | 17.3% | -2.2% | 19.5% |
| **KFRE 5%** | 19.4% | -3.6% | 23.0% |
| **KFRE 9%*** | 20.6% | -6.1% | 27.7% |

* Optimal threshold according to Youden Index

Abbreviations: KDIGO, Kidney Disease Improving Global Outcome; KFRE, Kidney Failure Risk Equation; SCREAM, Stockholm CREAtinine Measurements; NRI; Net Reclassification Index; NRI+; Net Reclassification Index for events; NRI-; Net Reclassification Index for non-events.

### **Table S7:** Sensitivity and Specificity for Non-North America KFRE and the SCREAM recalibrated KFRE across different thresholds.

|  | **Non-North America model** | | **SCREAM recalibrated model** | |
| --- | --- | --- | --- | --- |
| Threshold | Sensitivity | Specificity | Sensitivity | Specificity |
| 0.01 | 0.99 | 0.54 | 0.97 | 0.74 |
| 0.02 | 0.99 | 0.66 | 0.96 | 0.80 |
| 0.03 | 0.98 | 0.72 | 0.95 | 0.82 |
| 0.04 | 0.97 | 0.76 | 0.94 | 0.84 |
| 0.05 | 0.97 | 0.78 | 0.93 | 0.86 |
| 0.06 | 0.96 | 0.80 | 0.92 | 0.87 |
| 0.07 | 0.96 | 0.81 | 0.91 | 0.88 |
| 0.08 | 0.95 | 0.83 | 0.91 | 0.89 |
| 0.09 | 0.95 | 0.84 | 0.90 | 0.90 |
| 0.10 | 0.94 | 0.85 | 0.89 | 0.90 |
| 0.11 | 0.93 | 0.86 | 0.89 | 0.90 |
| 0.12 | 0.93 | 0.87 | 0.88 | 0.91 |
| 0.13 | 0.92 | 0.88 | 0.87 | 0.92 |
| 0.14 | 0.91 | 0.88 | 0.86 | 0.92 |
| 0.15 | 0.91 | 0.89 | 0.86 | 0.93 |
| 0.16 | 0.90 | 0.89 | 0.85 | 0.93 |
| 0.17 | 0.90 | 0.90 | 0.84 | 0.93 |
| 0.18 | 0.89 | 0.90 | 0.84 | 0.95 |
| 0.19 | 0.88 | 0.91 | 0.83 | 0.94 |
| 0.20 | 0.88 | 0.91 | 0.82 | 0.94 |

*Abbreviations: SCREAM, Stockholm CREAtinine Measurements.*

### **Table S8:** Sensitivity analysis considering one random observation per patient. Sensitivity and specificity of nephrologist-referral models for prediction of 5-year KRT risk.

***Panel A:*** *Performance metrics across models of nephrologist-referral*

| **KFRE referral model** | | | | | **Traditional criteria** | | | |
| --- | --- | --- | --- | --- | --- | --- | --- | --- |
|  | **Non-North American KFRE** | | **SCREAM recalibrated KFRE** | | **Swedish referral model** | | **Classic KDIGO referral model** | |
| Threshold | Sensitivity | Specificity | Sensitivity | Specificity | Sensitivity | Specificity | Sensitivity | Specificity |
| **3%** | 0.96 | 0.84 | 0.92 | 0.91 | 0.96 | 0.77 | 0.95 | 0.74 |
| **5%** | 0.94 | 0.88 | 0.90 | 0.94 |  |  |  |  |
| **9% (*)** | - | - | 0.87 | 0.96 |  |  |  |  |
| **15% (*)** | 0.88 | 0.95 | - | - |  |  |  |  |

* Optimal threshold according to Youden Index

***Panel B:*** *Direct comparison of performance between Swedish referral criteria/classical KDIGO referral criteria and of Non-North America KFRE/SCREAM Recalibrated KFRE*

|  | **KFRE referral model** | | | | | |
| --- | --- | --- | --- | --- | --- | --- |
|  | **Non-North American KFRE** | | | **SCREAM recalibrated KFRE** | | |
|  | Corresponding threshold | Sensitivity | Specificity | Corresponding threshold | Sensitivity | Specificity |
| **Compared to Swedish referral model given:** | | | | | | |
| Same sensitivity | 2.2% | 0.96 | 0.80 | 0.5% | 0.96 | 0.80 |
| Same specificity | 1.7% | 0.97 | 0.77 | 0.4% | 0.96 | 0.77 |
| **Compared to classic KDIGO referral model given:** | | | | | | |
| Same sensitivity | 4.1% | 0.95 | 0.87 | 0.9% | 0.95 | 0.84 |
| Same specificity | 1.4% | 0.97 | 0.74 | 0.3% | 0.97 | 0.74 |

***Panel B:*** *Direct comparison of performance between Non-North America KFRE referral criteria and CREAM Recalibrated KFRE*

|  | **SCREAM recalibrated KFRE** | | |
| --- | --- | --- | --- |
|  | Corresponding threshold | Sensitivity | Specificity |
| **Compared to Non-North American KFRE (3%) given:** | | | |
| Same sensitivity | 0.7% | 0.96 | 0.83 |
| Same specificity | 0.8% | 0.95 | 0.84 |
| **Compared to Non-North American KFRE (3%) given:** | | | |
| Same sensitivity | 1.3% | 0.94 | 0.77 |
| Same specificity | 1.6% | 0.94 | 0.88 |

Abbreviations: KDIGO, Kidney Disease Improving Global Outcome; KFRE, Kidney Failure Risk Equation; SCREAM, Stockholm CREAtinine Measurements.

### **Table S9:** Sensitivity analysis considering only one random observation per patients. Classification performance of different nephrologist-referral models for predicting the 5-year risk of KRT.

|  | **Observations eligible for referral** | | | **Observations non-eligible for referral** | | |
| --- | --- | --- | --- | --- | --- | --- |
| **Referral model** | **Number of observations (proportion)** | **True positives**  **(PPV)** | **False positives** | **Number of observations (proportion)** | **True negatives**  **(NPV)** | **False negatives** |
| **Swedish referral model** | 45 359 (23%) | 2 200  (5%) | 43 159 (95%) | 147 605  (77%) | 147 528  (99.9%) | 77  (0.1%) |
| **Classical KDIGO referral model** | 49 970  (26%) | 2187  (4%) | 47783  (96%) | 142 994  (74%) | 142 904  (99.9%) | 90  (0.1%) |
| **KFRE referral model** | | | | | | |
| - **Non-North American KFRE** | | | | | | |
| **KFRE 3%** | 30 636 (16%) | 2 185  (7%) | 28 451 (93%) | 162 328 (84%) | 162 236  (99.9%) | 92  (0.1%) |
| **KFRE 5%** | 22 527 (12%) | 2 158 (10%) | 20 369 (90%) | 170 437 (88%) | 170 318  (99.9%) | 119  (0.1%) |
| **KFRE 15% (*)** | 10 719  (6%) | 2 034 (19%) | 8 685 (81%) | 182 245  (94%) | 182 002  (99.9%) | 243  (0.1%) |
| - **SCREAM recalibrated KFRE** | | | | | | |
| **KFRE 3%** | 17 304  (9%) | 2 112 (12%) | 15 192 (88%) | 175 660  (91%) | 175 495  (99.9%) | 165  (0.1%) |
| **KFRE 5%** | 13 446  (7%) | 2 073 (15%) | 11 373 (85%) | 179 518  (93%) | 179 314  (99.9%) | 204  (0.1%) |
| **KFRE 9% (*)** | 9 778  (5%) | 2 018 (21%) | 7 760 (79%) | 183 186  (95%) | 182 927  (99.9%) | 259  (0.1%) |

* Optimal threshold according to Youden Index

Abbreviations: KDIGO, Kidney Disease Improving Global Outcome; KFRE, Kidney Failure Risk Equation; SCREAM, Stockholm CREAtinine Measurements.

**Table S10:** Sensitivity analysis considering only one random observation per patients. Reclassification performance of the KFRE referral model (panel A non-north American KFRE, Panel B SCREAM recalibrated KFRE) versus the Swedish and classic KDIGO referral models in the prediction of 5-year risk of KRT.

| **Panel A. Non-North American KFRE referral model** | | | | | | | |
| --- | --- | --- | --- | --- | --- | --- | --- |
|  | **KFRE 3%** | | **KFRE 5%** | | **KFRE 15*%** | |  |
|  | *No referral* | *Referral* | *No referral* | *Referral* | *No referral* | *Referral* | *Total* |
| **Swedish referral model** | | | | | | | |
| *No referral* | 139 173 (72%) | 8 432  (5%) | 142 634 (74%) | 4 971  (3%) | 147 037 (77%) | 568 (0.30%) | 147 605  (77%) |
| *Referral* | 23 155  (12%) | 22 204  (11%) | 27 803 (14%) | 17 556  (9%) | 35 208 (18%) | 10 151  (5%) | 45 359 (23%) |
| *Total* | 162 328 (84%) | 30 636 (16%) | 170 437 (88%) | 22 527 (12%) | 182 245  (94%) | 10 719  (6%) |  |
| **KDIGO referral model** | | | | | | | |
| *No referral* | 137 029  (71%) | 5 965  (3%) | 140 800  (73%) | 2 194 (1%) | 142 924  (74%) | 70 (0.04%) | 142 994  (74%) |
| *Referral* | 25 299  (13%) | 24 671 (13%) | 29 637  (15%) | 20 333 (11%) | 39 321  (20%) | 10 649 (6%) | 49 970  (26%) |
| *Total* | 162 328 (84%) | 30 636 (16%) | 170 437 (88%) | 22 527 (12%) | 182 245  (94%) | 10 719  (6%) |  |
| **Panel B. SCREAM recalibrated referral model** | | | | | | | |
|  | **KFRE 3%** | | **KFRE 5%** | | **KFRE 9*%** | |  |
|  | *No*  *referral* | *Referral* | *No*  *referral* | *Referral* | *No*  *referral* | *Referral* | *Total* |
| **Swedish referral model** | | | | | | | |
| *No referral* | 144 044  (75%) | 3 561  (2%) | 145 683  (76%) | 1 922 (0.998%) | 147 032  (76%) | 573  (0.300%) | 147 605  (77%) |
| *Referral* | 31 616  (16%) | 13 743  (7%) | 33 835  (17%) | 11 524 (6%) | 36 154  (19%) | 9 205  (5%) | 45 359 (23%) |
| *Total* | 175 660  (91%) | 17 304  (9%) | 179 518  (93%) | 13 446  (7%) | 183 186  (95%) | 9 778  (5%) |  |
| **KDIGO referral model** | | | | | | | |
| *No referral* | 142 374  (74%) | 684  (0.3%) | 142 780  (74%) | 214  (0.1%) | 142 942 (74%) | 52  (0.03%) | 142 994  (74%) |
| *Referral* | 33 350  (17%) | 16 620 (9%) | 36 738  (19%) | 13 232 (7%) | 40 244 (21%) | 9 726 (5%) | 49 970  (26%) |
| *Total* | 175 660  (91%) | 17 304  (9%) | 179 518  (93%) | 13 446  (7%) | 183 186  (95%) | 9 778  (5%) |  |

* Optimal threshold according to Youden Index

Abbreviations: KDIGO, Kidney Disease Improving Global Outcome; KFRE, Kidney Failure Risk Equation; SCREAM, Stockholm CREAtinine Measurements.

### **Table S11:** Sensitivity analysis considering only uACR observations. Sensitivity and specificity of nephrologist-referral models for prediction of 5-year KRT risk.

***Panel A:*** *Performance metrics across models of nephrologist-referral*

| **KFRE referral model** | | | | | **Traditional criteria** | | | |
| --- | --- | --- | --- | --- | --- | --- | --- | --- |
|  | **Non-North American KFRE** | | **SCREAM recalibrated KFRE** | | **Swedish referral model** | | **Classic KDIGO referral model** | |
| Threshold | Sensitivity | Specificity | Sensitivity | Specificity | Sensitivity | Specificity | Sensitivity | Specificity |
| **3%** | 0.98 | 0.64 | 0.95 | 0.76 | 0.99 | 0.55 | 0.98 | 0.53 |
| **5%** | 0.97 | 0.70 | 0.940 | 0.80 |  |  |  |  |
| **9% (*)** | - | - | 0.91 | 0.85 |  |  |  |  |
| **15% (*)** | 0.92 | 0.83 | - | - |  |  |  |  |

* Optimal threshold according to Youden Index

***Panel B:*** *Direct comparison of performance between Swedish referral criteria/classical KDIGO referral criteria and of Non-North America KFRE/SCREAM Recalibrated KFRE.*

|  | **KFRE referral model** | | | | | |
| --- | --- | --- | --- | --- | --- | --- |
|  | **Non-North American KFRE** | | | **SCREAM recalibrated KFRE** | | |
|  | Corresponding threshold | Sensitivity | Specificity | Corresponding threshold | Sensitivity | Specificity |
| **Swedish referral model** | | | | | | |
| Same sensitivity | 2.5% | 0.99 | 0.61 | 0.99 | 0.59 | 0.5% |
| Same specificity | 1.6% | 0.99 | 0.55 | 0.99 | 0.55 | 0.3% |
| **Classic KDIGO referral model** | | | | | | |
| Same sensitivity | 3.8% | 0.98 | 0.67 | 0.98 | 0.64 | 0.8% |
| Same specificity | 1.4% | 0.99 | 0.53 | 0.99 | 0.53 | 0.3% |

***Panel B:*** *Direct comparison of performance between Non-North America KFRE referral criteria and SCREAM Recalibrated KFRE.*

|  | **SCREAM recalibrated KFRE** | | |
| --- | --- | --- | --- |
|  | Corresponding threshold | Sensitivity | Specificity |
| **Compared to Non-North American KFRE (3%) given:** | | | |
| Same sensitivity | 0.6% | 0.98 | 0.61 |
| Same specificity | 0.8% | 0.98 | 0.64 |
| **Compared to Non-North American KFRE (3%) given:** | | | |
| Same sensitivity | 1.3% | 0.97 | 0.68 |
| Same specificity | 1.6% | 0.70 | 0.70 |

Abbreviations: KDIGO, Kidney Disease Improving Global Outcome; KFRE, Kidney Failure Risk Equation; SCREAM, Stockholm CREAtinine Measurements.

### **Table S12:** Sensitivity analysis considering only uACR observations. Classification performance of different nephrologist-referral models for predicting the 5-year risk of KRT.

|  | **Observations eligible for referral** | | | **Observations non-eligible for referral** | | |
| --- | --- | --- | --- | --- | --- | --- |
| **Referral model** | **Number of observations (proportion)** | **True positives**  **(PPV)** | **False positives** | **Number of observations (proportion)** | **True negatives**  **(NPV)** | **False negatives** |
| **Swedish referral model** | 212 640  (45%) | 30 000  (14%) | 182 640  (86%) | 262 204  (55%) | 261 829 (99.9%) | 375  (0.1%) |
| **Classic KDIGO referral model** | 221 233  (47%) | 29 890  (14%) | 191 343  (86%) | 253 611  (53%) | 253126  (99.8%) | 485  (0.2%) |
| **KFRE referral model** | | | | | | |
| - **Non-North American KFRE** | | | | | | |
| **KFRE 3%** | 176 356  (37%) | 29 957  (17.0%) | 146 399 (83%) | 298 488  (63%) | 298 070 (99.9%) | 418  (0.1%) |
| **KFRE 5%** | 148 910  (31%) | 29 693  (20%) | 119 217 (80%) | 325 934  (69%) | 325 252 (99.8%) | 682  (0.2%) |
| **KFRE 15% (*)** | 97 952  (21%) | 28 375 (29%) | 69 577  (71%) | 376 892  (79%) | 374 892  (99.5%) | 2000 (0.5%) |
| - **SCREAM recalibrated KFRE** | | | | | | |
| **KFRE 3%** | 128 043  (27%) | 29 238  (23%) | 98 805 (77%) | 346 801  (73%) | 345 664 (99.7%) | 1 137 (0.3%) |
| **KFRE 5%** | 110 988  (23%) | 28 812  (26%) | 82 176 (74%) | 363 856  (77%) | 362 293 (99.6%) | 1 563 (0.4%) |
| **KFRE 9% (*)** | 92 012  (19%) | 28 073  (31%) | 63 939 (69%) | 382 832  (81%) | 380 530 (99.4%) | 2302  (0.6%) |

* Optimal threshold according to Youden Index

Abbreviations: KDIGO, Kidney Disease Improving Global Outcome; KFRE, Kidney Failure Risk Equation; SCREAM, Stockholm CREAtinine Measurements.

**Table S13:** Sensitivity analysis considering only uACR observations. Reclassification performance of the KFRE referral model (panel A non-north American KFRE, Panel B SCREAM recalibrated KFRE) versus the Swedish and classic KDIGO referral models in the prediction of 5-year risk of KRT.

| **Panel A. Non-North American KFRE referral model** | | | | | | | |
| --- | --- | --- | --- | --- | --- | --- | --- |
|  | **KFRE 3%** | | **KFRE 5%** | | **KFRE 15*%** | |  |
|  | *No referral* | *Referral* | *No referral* | *Referral* | *No referral* | *Referral* | *Total* |
| **Swedish referral model** | | | | | | | |
| *No referral* | 237326  (50%) | 24878  (5%) | 246426  (52%) | 15778  (3%) | 259596  (54%) | 2608  (0.5%) | 262204  (55%) |
| *Referral* | 61162  (13%) | 151478  (32%) | 79508  (17%) | 133132  (28%) | 117296  (25%) | 95344  (20%) | 212640  (45%) |
| *Total* | 298488  (63%) | 176356  (37%) | 325934  (69%) | 148910  (31%) | 376892  (79%) | 97952  (21%) |  |
| **Classic KDIGO referral model** | | | | | | | |
| *No referral* | 233673  (49%) | 19938  (4%) | 244697  (51%) | 8914  (2%) | 253146  (53%) | 465  (0.1%) | 253 611  (53%) |
| *Referral* | 64815  (14%) | 156418  (33%) | 81237  (18%) | 139996 (29%) | 123746  (26%) | 97487  (21%) | 221 233  (47%) |
| *Total* | 298488  (63%) | 176356  (37%) | 325934  (69%) | 148910  (31%) | 376892  (79%) | 97952  (21%) |  |
| **SCREAM recalibrated model** | | | | | | | |
|  | **KFRE 3%** | | **KFRE 5%** | | **KFRE 9*%** | |  |
|  | *No*  *referral* | *Referral* | *No*  *referral* | *Referral* | *No*  *referral* | *Referral* | *Total* |
| **Swedish referral model** | | | | | | | |
| *No referral* | 250283  (53%) | 11921 (2%) | 255085  (54%) | 7119  (1%) | 259525  (55%) | 2679  (0.6%) | 262204  (55%) |
| *Referral* | 96518  (20%) | 116122 (25%) | 108771  (23%) | 103869 (22%) | 123307  (26%) | 89333  (19%) | 212640  (45%) |
| *Total* | 346801  (73%) | 128043  (27%) | 363856  (77%) | 110988  (23%) | 382832  (81%) | 92012  (19%) |  |
| **Classic KDIGO referral model** | | | | | | | |
| *No referral* | 250054  (52%) | 3557  (0.7%) | 252238  (53%) | 1373  (0.3%) | 253210 (53%) | 401  (0.08%) | 253 611  (53%) |
| *Referral* | 96747  (21%) | 124486 (26%) | 111618  (24%) | 109615 (23%) | 129622 (28%) | 91611 (19%) | 221 233  (47%) |
| *Total* | 346801  (73%) | 128043  (27%) | 363856  (77%) | 110988  (23%) | 382832  (81%) | 92012  (19%) |  |

* Optimal threshold according to Youden Index

Abbreviations: KDIGO, Kidney Disease Improving Global Outcome; KFRE, Kidney Failure Risk Equation; SCREAM, Stockholm CREAtinine Measurements.

### **Table S14:** Sensitivity analysis considering three different time periods. Sensitivity and specificity of nephrologist-referral models for prediction of 5-year KRT risk.

| **Inclusion period 2006 - 2010** | | | | | | | | |
| --- | --- | --- | --- | --- | --- | --- | --- | --- |
| **KFRE referral model** | | | | | **Traditional criteria** | | | |
|  | **Non-North American KFRE** | | **SCREAM recalibrated KFRE** | | **Swedish referral model** | | **Classic KDIGO referral model** | |
| Threshold | Sensitivity | Specificity | Sensitivity | Specificity | Sensitivity | Specificity | Sensitivity | Specificity |
| **3%** | 0.98 | 0.74 | 0.95 | 0.82 | 0.98 | 0.68 | 0.98 | 0.65 |
| **5%** | 0.97 | 0.79 | 0.94 | 0.82 |  |  |  |  |
| **9% (*)** | - | - | 0.90 | 0.90 |  |  |  |  |
| **15% (*)** | 0.91 | 0.89 | - | - |  |  |  |  |
| **Inclusion period 2011 - 2015** | | | | | | | | |
| **KFRE referral model** | | | | | **Traditional criteria** | | | |
|  | **Non-North American KFRE** | | **SCREAM recalibrated KFRE** | | **Swedish referral model** | | **Classic KDIGO referral model** | |
| Threshold | Sensitivity | Specificity | Sensitivity | Specificity | Sensitivity | Specificity | Sensitivity | Specificity |
| **3%** | 0.98 | 0.71 | 0.95 | 0.81 | 0.98 | 0.64 | 0.98 | 0.62 |
| **5%** | 0.97 | 0.77 | 0.93 | 0.85 |  |  |  |  |
| **9% (*)** | - | - | 0.90 | 0.89 |  |  |  |  |
| **15% (*)** | 0.91 | 0.88 | - | - |  |  |  |  |
| **Inclusion period 2016 - 2021** | | | | | | | | |
| **KFRE referral model** | | | | | **Traditional criteria** | | | |
|  | **Non-North American KFRE** | | **SCREAM recalibrated KFRE** | | **Swedish referral model** | | **Classic KDIGO referral model** | |
| Threshold | Sensitivity | Specificity | Sensitivity | Specificity | Sensitivity | Specificity | Sensitivity | Specificity |
| **3%** | 0.98 | 0.73 | 0.95 | 0.83 | 0.98 | 0.66 | 0.98 | 0.66 |
| **5%** | 0.97 | 0.79 | 0.94 | 0.87 |  |  |  |  |
| **9% (*)** | - | - | 0.91 | 0.90 |  |  |  |  |
| **15% (*)** | 0.91 | 0.89 | - | - |  |  |  |  |

* Optimal threshold according to Youden Index

Abbreviations: KDIGO, Kidney Disease Improving Global Outcome; KFRE, Kidney Failure Risk Equation; SCREAM, Stockholm CREAtinine Measurements.

### **Table S15:** TRIPO checklist

| **Section/Topic** | **Item** | **Checklist Item** | **Section/Paragraph** |
| --- | --- | --- | --- |
| **Title and abstract** | | | |
| Title | 1 | Identify the study as developing and/or validating a multivariable prediction model, the target population, and the outcome to be predicted. | Title |
| Abstract | 2 | Provide a summary of objectives, study design, setting, participants, sample size, predictors, outcome, statistical analysis, results, and conclusions. | Abstract |
| **Introduction** | | | |
| Background and objectives | 3a | Explain the medical context (including whether diagnostic or prognostic) and rationale for developing or validating the multivariable prediction model, including references to existing models. | Introduction, Para 1-3 |
|  | 3b | Specify the objectives, including whether the study describes the development or validation of the model or both. | Introduction, Para 4 |
| **Methods** | | | |
| Source of data | 4a | Describe the study design or source of data (e.g., randomized trial, cohort, or registry data), separately for the development and validation data sets, if applicable. | Methods, Para 1-2 |
|  | 4b | Specify the key study dates, including start of accrual; end of accrual; and, if applicable, end of follow-up. | Methods, Para 3 |
| Participants | 5a | Specify key elements of the study setting (e.g., primary care, secondary care, general population) including number and location of centres. | Methods, Para 1-2 |
|  | 5b | Describe eligibility criteria for participants. | Methods, Para 3 |
|  | 5c | Give details of treatments received, if relevant. | n/a |
| Outcome | 6a | Clearly define the outcome that is predicted by the prediction model, including how and when assessed. | Methods, Para 9 |
|  | 6b | Report any actions to blind assessment of the outcome to be predicted. | n/a |
| Predictors | 7a | Clearly define all predictors used in developing or validating the multivariable prediction model, including how and when they were measured. | Methods, Para 4-5 |
|  | 7b | Report any actions to blind assessment of predictors for the outcome and other predictors. | n/a |
| Sample size | 8 | Explain how the study size was arrived at. | Methods, Para 3, Supplementary material |
| Missing data | 9 | Describe how missing data were handled (e.g., complete-case analysis, single imputation, multiple imputation) with details of any imputation method. | Methods, Para 3 |
| Statistical analysis methods | 10c | For validation, describe how the predictions were calculated. | Methods, Para 12,  Supplementary material |
|  | 10d | Specify all measures used to assess model performance and, if relevant, to compare multiple models. | Methods Para 5-6-7 |
|  | 10e | Describe any model updating (e.g., recalibration) arising from the validation, if done. | Methods Para 11 |
| Risk groups | 11 | Provide details on how risk groups were created, if done. | Methods, Para 12 |
| Development vs. validation | 12 | For validation, identify any differences from the development data in setting, eligibility criteria, outcome, and predictors. | Results, Para 12 |
| **Results** | | | |
| Participants | 13a | Describe the flow of participants through the study, including the number of participants with and without the outcome and, if applicable, a summary of the follow-up time. A diagram may be helpful. | Results, Para 1, Figure S1 |
|  | 13b | Describe the characteristics of the participants (basic demographics, clinical features, available predictors), including the number of participants with missing data for predictors and outcome. | Results, Para 1, Table 1 |
|  | 13c | For validation, show a comparison with the development data of the distribution of important variables (demographics, predictors and outcome). | Results, Para 1, Table 1 |
| Model performance | 16 | Report performance measures (with CIs) for the prediction model. | Results, Para 2-3, Table S4 |
| Model-updating | 17 | If done, report the results from any model updating (i.e., model specification, model performance). | Results, Para 2, Table 2-3-4, Figures 1-2, Supplementary material |
| **Discussion** | | | |
| Limitations | 18 | Discuss any limitations of the study (such as nonrepresentative sample, few events per predictor, missing data). | Discussion, Para 7 |
| Interpretation | 19a | For validation, discuss the results with reference to performance in the development data, and any other validation data. | Discussion, Para 2-3 |
|  | 19b | Give an overall interpretation of the results, considering objectives, limitations, results from similar studies, and other relevant evidence. | Discussion, Para 1-2-3 |
| Implications | 20 | Discuss the potential clinical use of the model and implications for future research. | Discussion, Para 7 |
| **Other information** | | | |
| Supplementary information | 21 | Provide information about the availability of supplementary resources, such as study protocol, Web calculator, and data sets. | Methods, Para 15 |
| Funding | 22 | Give the source of funding and the role of the funders for the present study. | Submission information, Funding |

**Figure S1:** Study flowchart.

Stockholm residents accessing healthcare during 2006-2021, with at least one creatinine and one albuminuria test on the same date or within 12 months

n= 945 212 participants; n=4 000 447 observations

Exclude participants who, at first observation have:

- Age < 18 (n= 69 250)
- KRT (n=1 281)
- eGFR>60 ml/min/1.73 m^2^ (n=681 597)

Eligible participants for study

n=193 084 participants; n=917 696 observations

Exclude participants who die within one day of cohort inclusion (n=70)

Final Individuals included in the study

n=192 964 participants; n=887 388 observations

*Abbreviations: eGFR, estimated glomerular filtration rate; KRT, kidney replacement therapy.*

**Figure S2:** Cumulative incidence curves of KRT events across KFRE risk groups. **
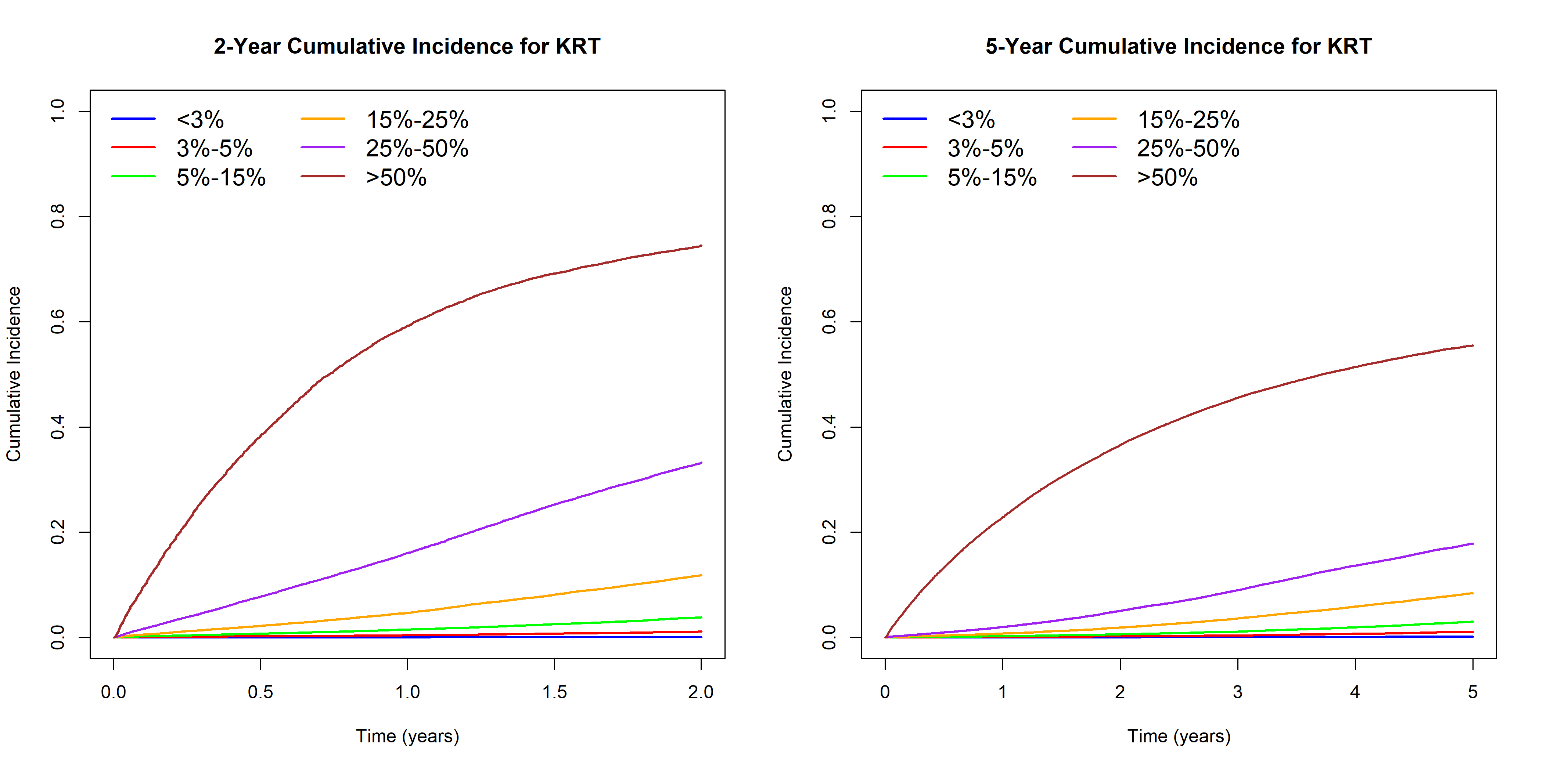
**

*Risk groups were defined according to previously proposed risk groups of <3%, 3% to <5%, 5% to <15%, 15% to <25%, 25% to <50% and ≥50%. Risk categorization was based on the risk Non- North America KFRE. Left plot: 2 years KRT risk. Right plot: 5 years KRT risk.*

*Abbreviations: KRT, kidney replacement therapy.*

**Figure S3:** Calibration plot of expected versus observed 2-year KRT risk, considering the non-North America calibrated KFRE (panel A) and the SCREAM recalibrated KFRE (panel B).


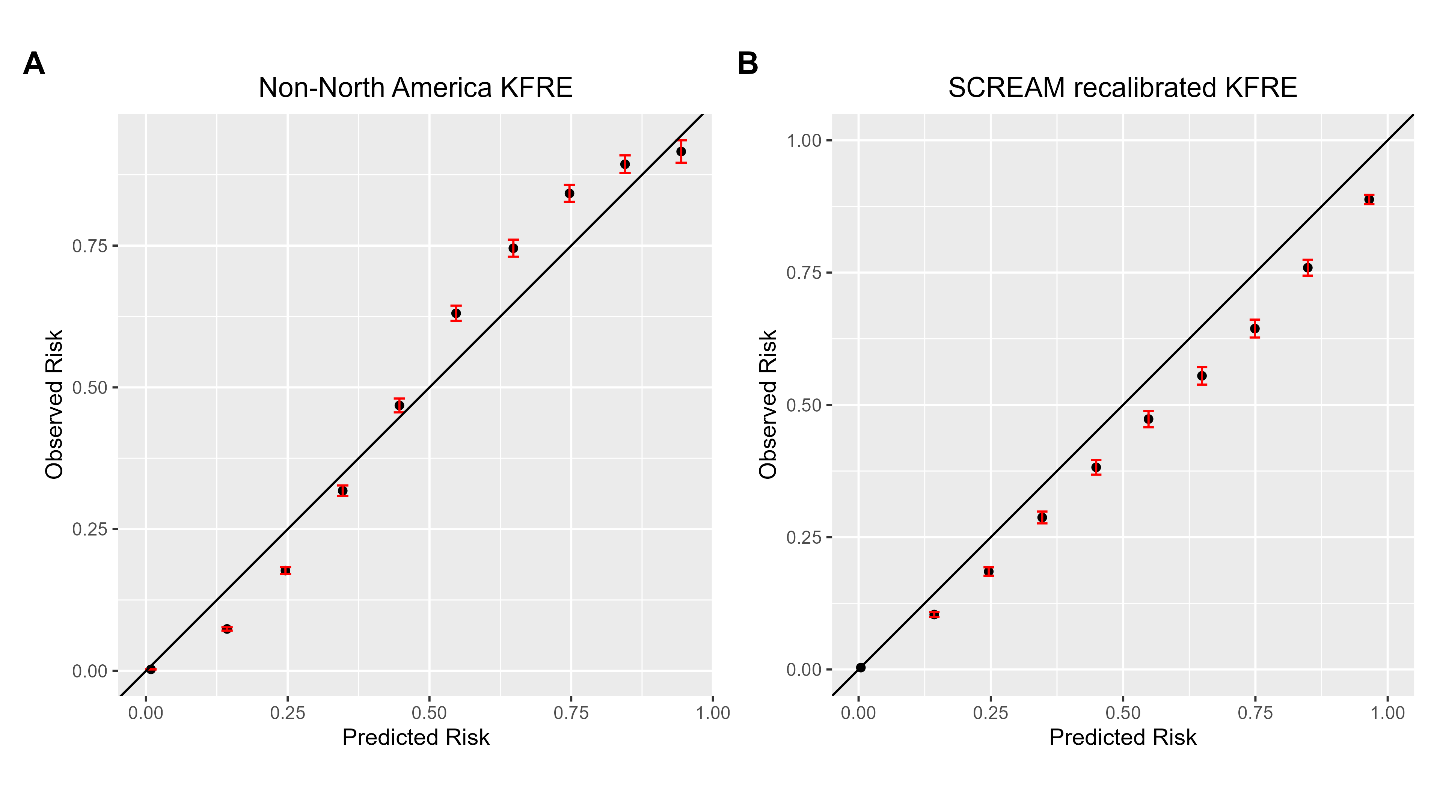


*Groups are split into 10% of predicted risk. The black dots represent the predicted and observed risk for each group. The red vertical lines represent the 95% CIs. The black line indicates perfect calibration.*

*Abbreviations: KFRE, Kidney Failure Risk Equation; SCREAM, Stockholm CREAtinine Measurements.*

**Figure S4:** Calibration plot of expected versus observed 5-year KRT risk for risk<20%, considering the non-North America calibrated KFRE (panel A) and the SCREAM recalibrated KFRE (panel B).
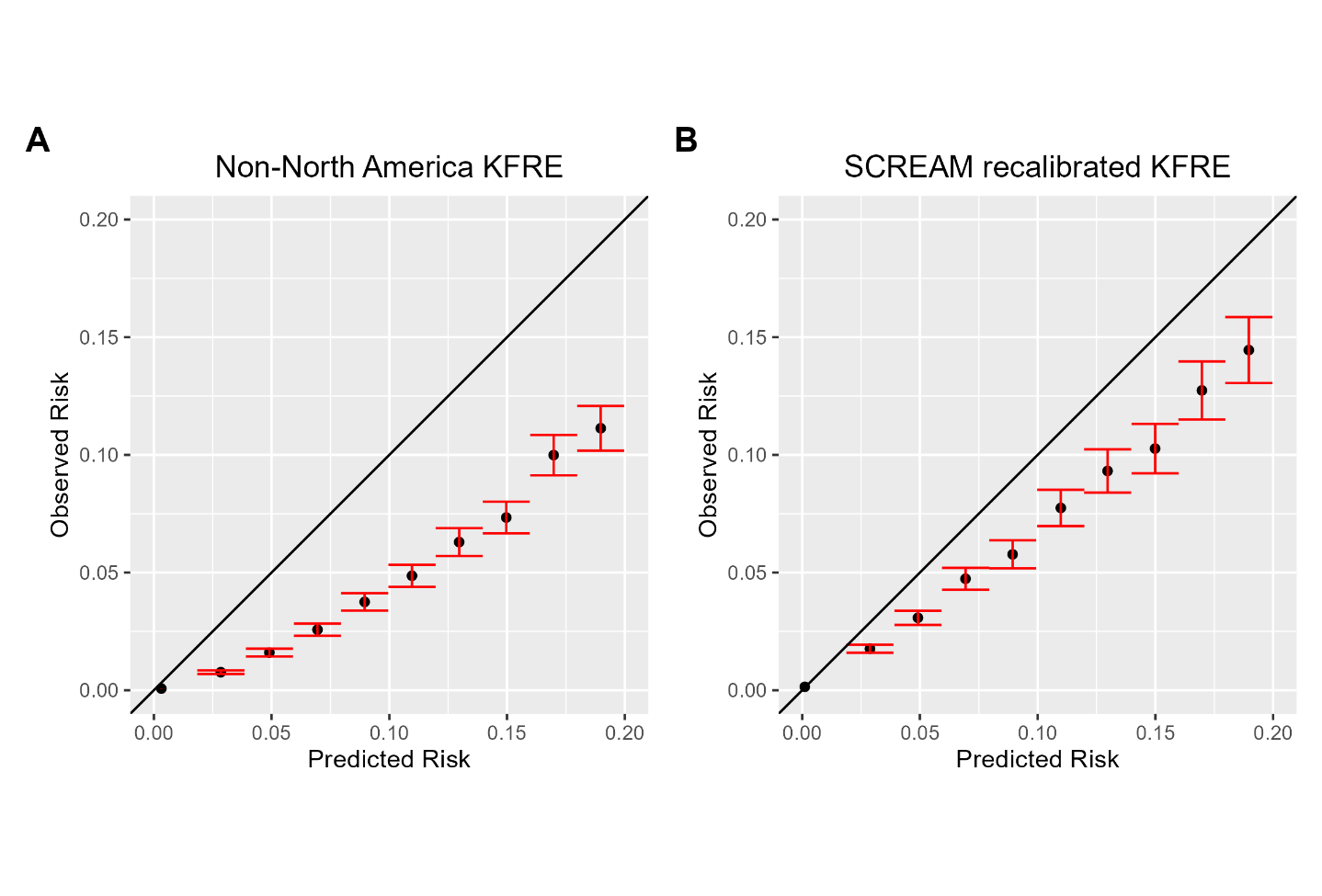


*The black dots represent the predicted and observed risk for each group. The red vertical lines represent the 95% CIs. The black line indicates perfect calibration.*

*Abbreviations: KFRE, Kidney Failure Risk Equation; SCREAM, Stockholm CREAtinine Measurements.*

**Figure S5:** Calibration plot of expected versus observed 2-year KRT risk for risk<20%, considering the non-North America calibrated KFRE (panel A) and the SCREAM recalibrated KFRE (panel B).
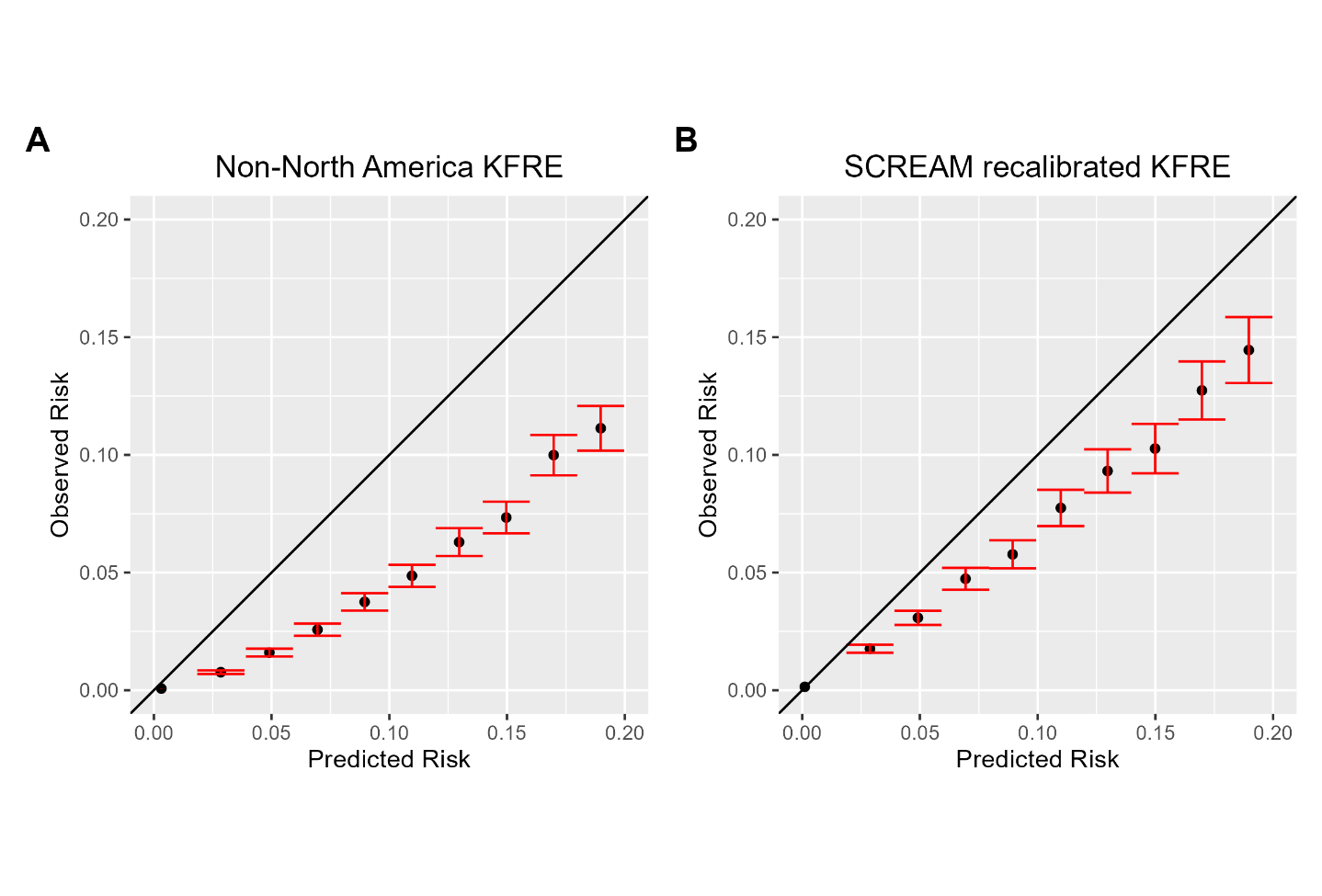


*The black dots represent the predicted and observed risk for each group. The red vertical lines represent the 95% CIs. The black line indicates perfect calibration.*

*Abbreviations: KFRE, Kidney Failure Risk Equation; SCREAM, Stockholm CREAtinine Measurements.*

### **Figure S6**: Time-varying Receiver Operator Characteristic (ROC) curves for the non-North America calibrated KFRE and the SCREAM recalibrated KFRE in the prediction of 5-year KRT risk.


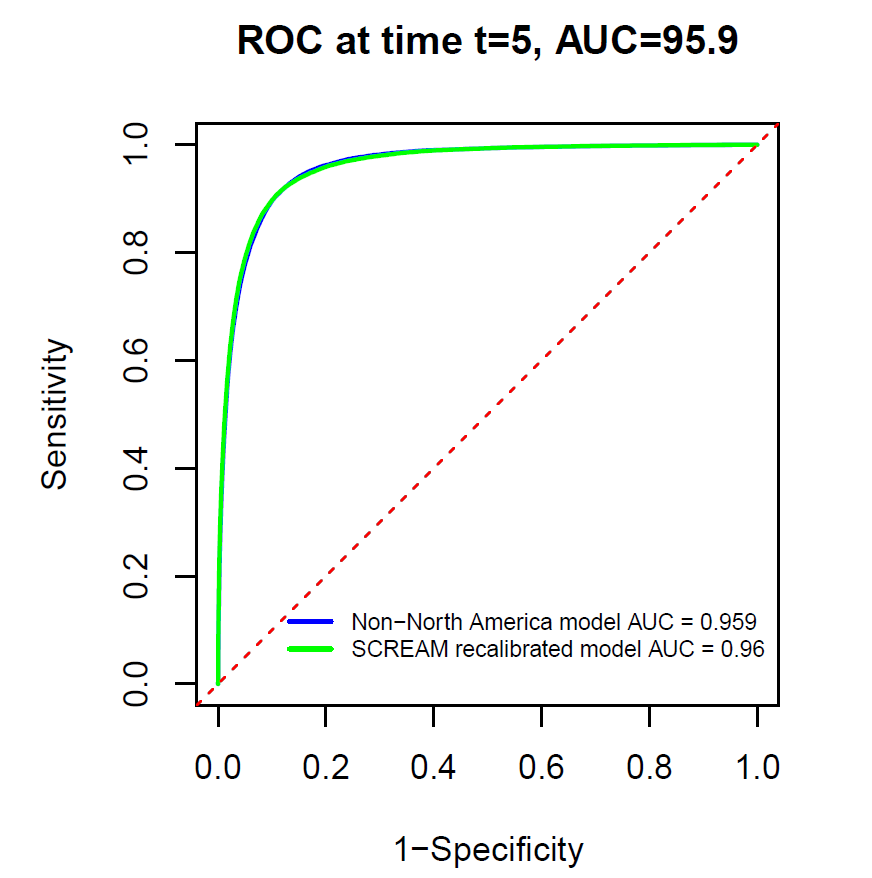


*Abbreviations: AUC, Area Under the Curve; SCREAM, Stockholm CREAtinine Measurements.*

**Figure S7:** Sensitivity analysis considering one random observation per patient. Decision curve analyses comparing differences in net benefit across various nephrologist-referral models for predicting the 5-year risk of KRT.


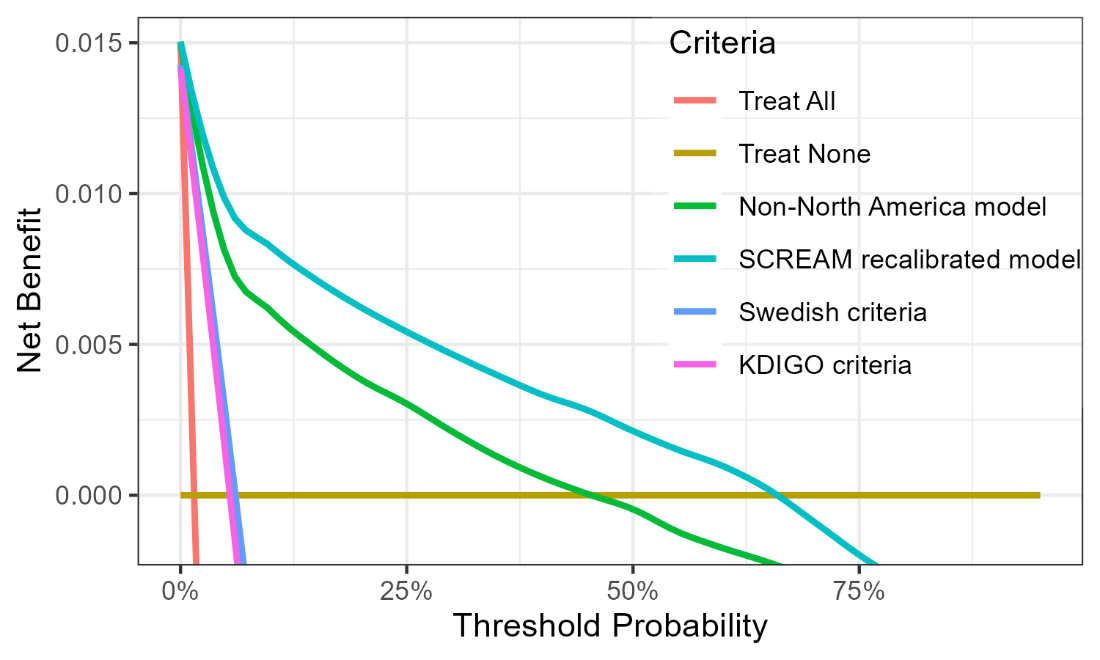


Threshold probabilities refer to the point at which a clinician would opt for treatment; so lower thresholds represent the clinical setting where the clinician is more concerned about missing true positives and therefore is willing to act even if the probability of the outcome is low. While high threshold probabilities mean that the clinician is more concerned about avoiding false positives and so clinician will only acts when there is a high probability of the outcome.

Abbreviations: KDIGO, Kidney Disease Improving Global Outcome; KFRE, Kidney Failure Risk Equation; SCREAM, Stockholm CREAtinine Measurements.

**Figure S8:** Sensitivity analysis considering only uACR observations. Decision curve analyses comparing differences in net benefit across various nephrologist-referral models for predicting the 5-year risk of KRT.


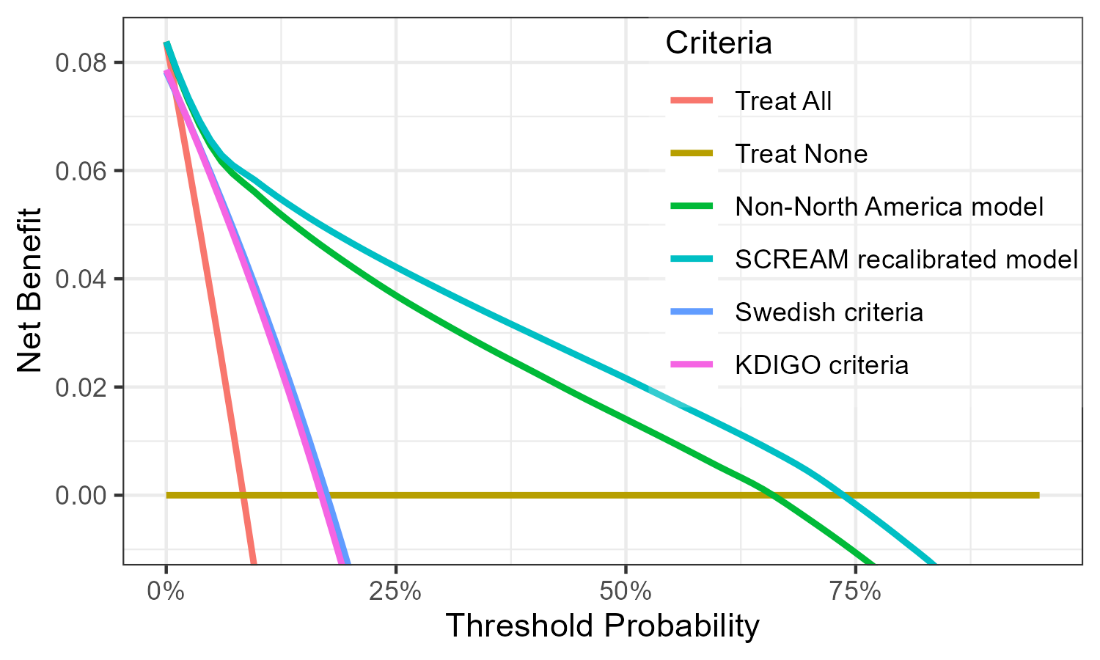


Threshold probabilities refer to the point at which a clinician would opt for treatment; so lower thresholds represent the clinical setting where the clinician is more concerned about missing true positives and therefore is willing to act even if the probability of the outcome is low. While high threshold probabilities mean that the clinician is more concerned about avoiding false positives and so clinician will only acts when there is a high probability of the outcome.

Abbreviations: KDIGO, Kidney Disease Improving Global Outcome; KFRE, Kidney Failure Risk Equation; SCREAM, Stockholm CREAtinine Measurements.

### **Appendix 1:** Non-north American model and SCREAM recalibrated model

**2 years models:**

Non-North American KFRE 🡪 1 - 0.9832^exp (-0.2201 * (age/10 – 7.036) + 0.2467 * (male – 0.5642) – 0.5567 * (eGFR/5 – 7.222) + 0.4510 * (logACR – 5.137))

SCREAM recalibrated KFRE🡪 1 - 0.9995581^exp (-0.365929 * (age/ 10 - 7.49) + 0.330359 * (male - 0.5037) - 1.086959 * (eGFR/5 – 8.63) + 0.441163 * ((logACR) – 5.62))

**5 years models:**

Non-North American KFRE 🡪 1 - 0.9365^exp (-0.2201 * (age/10 – 7.036) + 0.2467 * (male – 0.5642) – 0.5567 * (eGFR/5 – 7.222) + 0.4510 * (logACR – 5.137))

SCREAM recalibrated KFRE 🡪 1 - 0.9931158^exp (-0.368904 * (age/ 10 - 7.49) + 0. 322899 * (male - 0.5037) - 0. 796221 * (eGFR /5 – 8.63) + 0. 450980 * ((logACR) – 5.62))
